# Whole genome sequencing and phylogenetic characterisation of rabies virus strains from Moldova and north-eastern Romania

**DOI:** 10.1371/journal.pntd.0011446

**Published:** 2023-07-06

**Authors:** Mihaela Anca Dascalu, Evelyne Picard-Meyer, Emmanuelle Robardet, Alexandre Servat, Serghei Arseniev, Oxana Groza, Nicolae Starciuc, Vlad Vuta, Florica Barbuceanu, Oana Irina Tanase, Florentina Daraban Bocaneti, Helene Quenault, Edouard Hirchaud, Yannick Blanchard, Elena Velescu, Florence Cliquet

**Affiliations:** 1 Department of Public Health, Faculty of Veterinary Medicine, Iasi University of Life Sciences "Ion Ionescu de la Brad", Mihail Sadoveanu Alley, Romania; 2 ANSES, Nancy Laboratory for Rabies and Wildlife, WHO Collaborating Centre for Research and Management in Zoonoses Control, OIE Reference Laboratory for Rabies, European Union Reference Laboratory for Rabies, European Union Reference Laboratory for Rabies Serology, Technopôle Agricole et Vétérinaire, Malzéville, France; 3 Republican Center of Veterinary Diagnostic, Chisinau, Moldova; 4 Faculty of Veterinary Medicine, State Agrarian University, Chisinau, Moldova; 5 Institute for Diagnosis and Animal Health, OIE Reference Laboratory for Rabies, Bucharest, Romania; University of Agronomic Study and Veterinary Medicine, Faculty of Veterinary Medicine, Bucharest, Romania; 6 ANSES, Nancy Ploufragan-Plouzané-Niort Laboratory, Viral Genetics and Biosafety Unit, Technopôle Agricole et Vétérinaire, Malzéville, France; University of São Paulo, BRAZIL

## Abstract

**Background:**

Rabies is the oldest fatal zoonotic disease recognised as a neglected tropical disease and is caused by an RNA virus belonging to the genus *Lyssavirus*, family *Rhabdoviridae*.

**Methodology/Principal findings:**

A deep molecular analysis was conducted on full-length nucleoprotein (N) gene and whole genome sequences of rabies virus from 37 animal brain samples collected between 2012 and 2017 to study the circulation of rabies virus (RABV) variants. The overall aim was to better understand their distribution in Moldova and north-eastern Romania. Both Sanger and high throughput sequencing on Ion Torrent and Illumina platforms were performed. Phylogenetic analysis of the RABV sequences from both Moldova and Romania revealed that all the samples (irrespective of the year of isolation and the species) belonged to a single phylogenetic group: north-eastern Europe (NEE), clustering into three assigned lineages: RO#5, RO#6 and RO#7.

**Conclusions/Significance:**

High throughput sequencing of RABV samples from domestic and wild animals was performed for the first time for both countries, providing new insights into virus evolution and epidemiology in this less studied region, expanding our understanding of the disease.

## Author summary

High throughput sequencing has become a powerful tool for the epidemiological study of the rabies virus. Considering the lack of information from the two geographic areas included in the present research, a collection of samples from 2012 to 2017 from both domestic and wild animals positive for rabies was used to study the circulation of rabies virus variants. The aim was to better understand their distribution in Moldova and north-eastern Romania, two neighbouring countries. Sanger and high throughput sequencing on Ion Torrent and Illumina platforms were used. A single phylogenetic group, revealing the common past evolutionary history of the species, was identified. The phylogenetic group is called north-eastern Europe (NEE) and samples clustered into three assigned lineages RO#5, RO#6 and RO#7. High throughput sequencing on samples from both domestic and wild animals was performed for the first time for both countries, providing new insights into RABV evolution and epidemiology in these areas and expanding our understanding of the disease.

## Introduction

Rabies is the oldest disease recognised as a neglected tropical disease. It is zoonotic and almost always has a fatal outcome in humans if not prevented by timely post-exposure prophylaxis. Rabies is caused by an RNA virus belonging to the genus *Lyssavirus*, family *Rhabdoviridae*. Despite the availability of effective and safe vaccines for humans and animals, rabies still represents a global threat, transmitted by both domestic and wild animals [1].

Over the years, molecular studies of classical rabies virus (RABV) have broadened our understanding of the diversity of RABV worldwide [2]. Initial molecular epidemiological studies of RABV tended to focus on small targeted regions of the genome, such as the nucleoprotein (N) and glycoprotein (G) genes [3].

In recent years, high throughput sequencing (HTS) has been increasingly used, creating powerful opportunities for epidemiological studies for both RABV [4] and additional virus species within the *Lyssavirus* genus, which currently includes 17 species recognised by the International Committee on Taxonomy of Viruses (ICTV) [5].

The large amount of sequence data produced rapidly and cost-efficiently by HTS has considerably improved our understanding of rabies molecular epidemiology and evolution, with a significant impact on our knowledge of genus *Lyssavirus* taxonomy [6]. HTS has also revolutionised phylogeographic studies [7], allowing us to investigate patterns of transmission and spread [4], and to reveal genetic differences among RABV strains that may influence host tropism and pathogenicity [8].

Moldova (33.846 km^2^), located in south-eastern Europe, shares borders with Ukraine to the north, east and south and Romania to the west. From 2010 to 2021, epidemiological data showed increasing incidence of rabies among domestic animals and significantly lower incidence in wild animals [9]; this trend was also observed in other countries such as Ukraine [10], Russia and Turkey [11]. According to the World Organization for Animal Health (WOAH) and Rabies Bulletin Europe [12], between 2010 and 2021, a total of 1,052 positive cases were reported in Moldova, out of which 789 (75%) were found in domestic and 263 (25%) in wild animals. Since 2020, Moldova has received 100% European Union (EU) funding support for the implementation of oral rabies vaccination (ORV) campaigns, which are paramount for eliminating rabies in wildlife populations [13,14].

Romania (238.397 km^2^), the second country included in our study, is also located in south-eastern Europe and shares land borders with Ukraine to the north and east, Moldova to the east, Bulgaria to the south, Serbia to the south-west and Hungary to the west and open to the Black Sea to the south-east. It is considered to have one of the highest incidences of rabies in animals among European countries [15]. An epidemiological analysis undertaken between 2010 (before the implementation of ORV campaigns in foxes) and 2021 revealed a total of 1,869 positive rabies cases throughout the territory of Romania, out of which 1,302 (69.66%) were reported in wild and 567 (30.34%) in domestic animals, unlike the situation reported in neighbouring Moldova [11,16,17]. The incidence of rabies registered in domestic and wild animals decreased once Romania joined the European Union in 2007. The country has received EU financial support for wildlife rabies control by implementing ORV campaigns in foxes. Additionally, most of the positive cases reported between 2016 and 2021 were located in north-eastern Romania, close to the borders with Ukraine and Moldova, where rabies cases are still high [11,16,18]. ORV campaigns and compulsory pet vaccination have not yet eliminated rabies throughout Romania, where the virus is still maintained in fox populations.

The objective of this study was to conduct a deep molecular analysis on various full-length N gene and whole genome sequences of RABV from both domestic and wild animals positive for rabies from a collection of samples from 2012 to 2017. The aim was to study the circulation of RABV variants and better understand their distribution in Moldova and north-eastern Romania.

## Methods

### Animal samples

**Fig 1:**
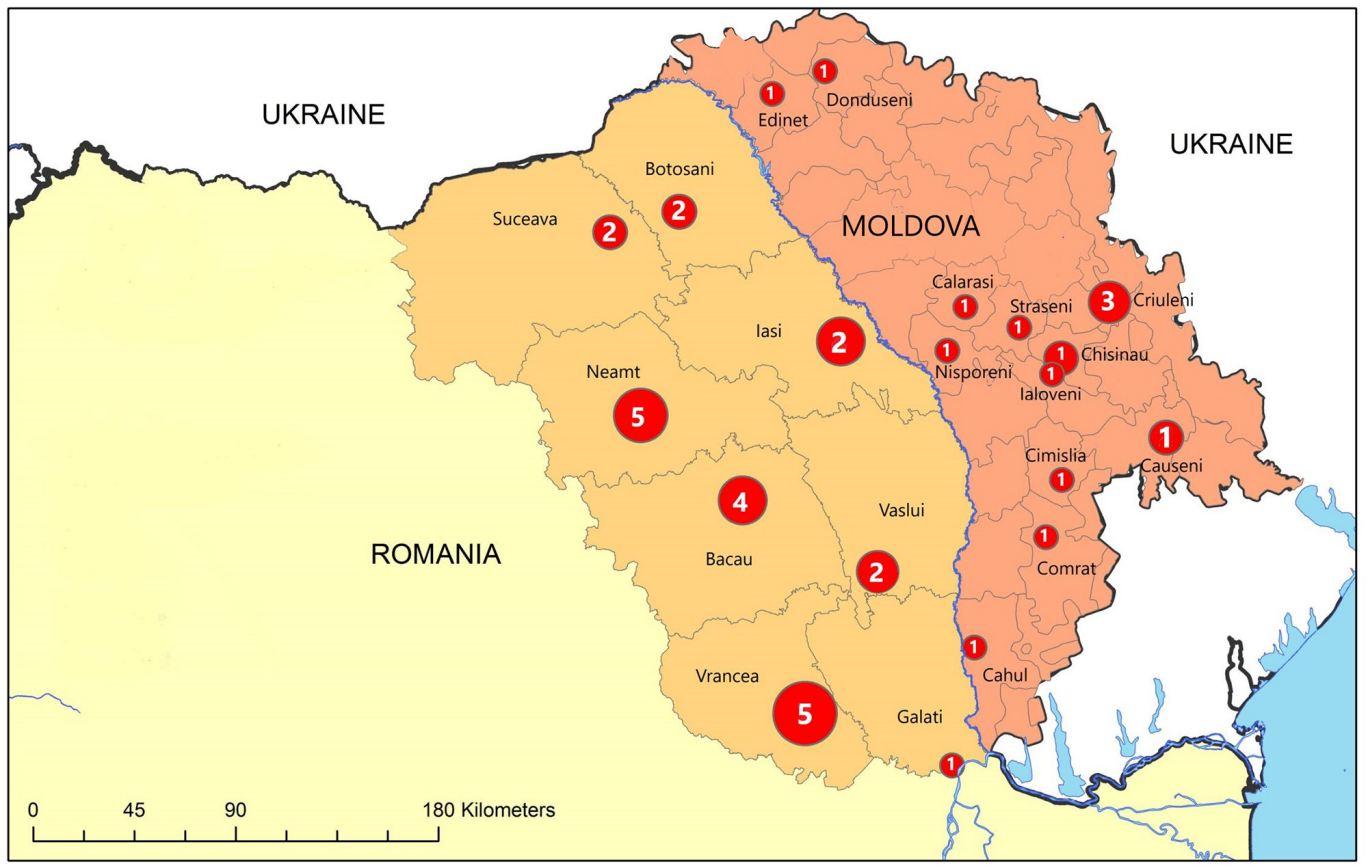
Map of Moldova and north-eastern Romania. The red circles represent the geographical origin of the samples used in the study. The circle sizes correspond to the number of tested samples (enlarge to view). The map depicted in Fig 1 is originally and was created using authorised license of ArcGIS, version 10.5.1.

In total, 37 animal brain samples were used in this study. Fourteen samples were collected across the whole territory of Moldova and 23 from north-eastern Romania (Fig 1).

The 14 brain samples from Moldova tested in this study (one wild and 13 domestic animal samples) were provided by the Republican Center of Veterinary Diagnostic (RCVD) in Chisinau. Samples were collected between 2016 and 2017 from the north (Donduseni, Edinet), south (Comrat, Cimislia), south-west (Cahul), south-east (Causeni), centre (Chisinau, Ialoveni, Straseni), centre-east (Criuleni) and centre-west of the country (Calarasi, Nisporeni).

The 23 brain samples (12 wild and 11 domestic animal samples) from north-eastern Romania were provided by the Sanitary Veterinary and Food Safety Directorate (SVFSD) from Iasi, Neamt, Vaslui, Bacau, Galati and Vrancea counties, as well as by the Institute for Diagnosis and Animal Health (IDAH) in Bucharest.

Table 1 shows the geographical origin of the samples, both from Moldova and north-eastern Romania, host species, code samples, year of isolation and the source of each sample.

**Table 1:** Characteristics of the brain samples used in the study from Moldova and north-eastern Romania.

No.	Country	County / GPS	Host species	Sample code	Year of isolation	Source
1	Romania	Vaslui 46°13′N 27°40′E	Cat	#DR1017	2014	*SVFSD VS
2	Romania	Vrancea 45.79°N 26.97°E	Red fox	#DR1019	2014	SVFSD VN
3	Romania	Vrancea 45.79°N 26.97°E	Red fox	DR1020	2013	SVFSD VN
4	Romania	Vrancea 45.79°N 26.97°E	Wolf	#DR1021	2014	SVFSD VN
5	Romania	Vrancea 45.79°N 26.97°E	Cow	DR1022	2013	SVFSD VN
6	Romania	Bacau 46°35′0″N 26°55′0″E	Red fox	#DR1024	2012	SVFSD BC
7	Romania	Bacau 46°35′0″N 26°55′0″E	Red fox	#DR1025	2012	SVFSD BC
8	Romania	Bacau 46°35′0″N 26°55′0″E	Dog	#DR1026	2012	SVFSD BC
9	Romania	Bacau 46°35′0″N 26°55′0″E	Deer	#DR1027	2012	SVFSD BC
10	Romania	Iasi 47°9′44″N 27°35′20″E	Cow	DR1030	2013	SVFSD IS
11	Romania	Galati 45°26′22″N 28°2′4″E	Cow	#DR1031	2015	SVFSD GL
12	Romania	Neamt 46°58′N 26°24′E	Dog	DR1032	2012	SVFSD NT
13	Romania	Neamt 46°58′N 26°24′E	Red fox	DR1033	2013	SVFSD NT
14	Romania	Neamt 46°58′N 26°24′E	Red fox	#DR1034	2013	SVFSD NT
15	Romania	Neamt 46°58′N 26°24′E	Cow	#DR1035	2012	SVFSD NT
16	Romania	Neamt 46°58′N 26°24′E	Dog	#DR1036	2013	SVFSD NT
17	Romania	Vaslui 46°13′N 27°40′E	Red fox	#DR1187	2014	SVFSD VS
18	Moldova	Chisinau 47°01′0″N 28°52′0″E	Goat	#DR1198	2016	*RCVD
19	Moldova	Criuleni 47°13′02″N 29°09′05″E	Dog	#DR1200	2016	RCVD
20	Moldova	Calarasi 47°15′N 28°18′E	Cow	DR1201	2016	RCVD
21	Romania	Vrancea 45.79°N 26.97°E	Red fox	#DR1331	2016	IDAH
22	Romania	Suceava 47°39′5″N 26°15′20″E	Cow	DR1332	2016	IDAH
23	Romania	Suceava 47°39′5″N 26°15′20″E	Red fox	#DR1333	2016	IDAH
24	Romania	Botoșani 47°44′N 26°41′E	Cow	DR1334	2016	IDAH
25	Romania	Iasi 47°9′44″N 27°35′20″E	Red fox	#DR1335	2016	IDAH
26	Romania	Botosani 47°44′N 26°41′E	Cow	DR1336	2016	IDAH
27	Moldova	Cahul 45°54′19″N 28°11′46″E	Cat	DR1343	2016	RCVD
28	Moldova	Criuleni 47°13′02″N 29°09′05″E	Cow	#DR1345	2016	RCVD
29	Moldova	Edinet 48°10′14″N 27°18′16″E	Cow	DR1347	2016	RCVD
30	Moldova	Comrat 46°18′01″N 28°39′26″E	Cat	#DR1348	2017	RCVD
31	Moldova	Nisporeni 47°5′0″N 28°11′0″E	Cow	#DR1349	2016	RCVD
32	Moldova	Cimislia 46°30′0″N 28°48′0″E	Cow	#DR1350	2016	RCVD
33	Moldova	Donduseni 48°13′N 27°35′E	Cat	#DR1351	2016	RCVD
34	Moldova	Criuleni 47°13′02″N 29°09′05″E	Cow	DR1352	2016	RCVD
35	Moldova	Causeni 46°38′N 29°24′E	Cow	DR1353	2016	RCVD
36	Moldova	Straseni 47°08′N 28°36′E	Cow	DR1356	2016	RCVD
37	Moldova	Ialoveni 46°57′N 28°47′E	Ferret	DR1357	2016	RCVD

(*SVFSD: Sanitary Veterinary and Food Safety Directorate; *RCVD: Republican Center of Veterinary Diagnostic; *IDAH: Institute for Diagnosis and Animal Health; VS: Vaslui county; VN: Vrancea county; BC: Bacau county; IS: Iasi county; GL: Galati county; NT: Neamt county; #: samples selected for whole genome sequencing of rabies virus—HTS)

### Rabies diagnosis

#### Direct fluorescent antibody (DFA) testing and cell culture tests

All samples used in this study were first studied by DFA testing and by mouse inoculation test (MIT) for the presence of rabies antigen and infectious rabies virus in the regional laboratories of Romania and Moldova, respectively. Before molecular characterisation performed in the present study, all samples were re-tested at the Nancy Laboratory for rabies and wildlife in France (during the period 2015–2017) using routine rabies diagnostic techniques: DFA and cell culture tests, as described in the WOAH Manual of diagnostic tests [19].

### RNA extraction

The preparation of RNA extractions, PCR reagents and mixtures was performed in separate rooms, and all the necessary safety measures were respected to avoid cross-contamination.

#### RNA extraction for samples subjected to conventional RT-PCR and Sanger sequencing

RNA was extracted from 200 μL of clarified supernatant of a 10% (w/v) brain suspension for each of the 37 brain samples used in the study (Table 1). A 10% brain tissue suspension was prepared by adding 9 mL of Dulbecco’s Modified Eagle’s Medium (DMEM) to a tube containing 1 g of the brain sample. Following a homogenisation step and centrifugation for 15 minutes at 3000 rpm/min, a volume of 200 μL of supernatant was collected and mixed with 200 μL of viral lysis buffer (Invitrogen, Villebon-sur-Yvette, France), as recommended by the manufacturer for RNA extraction. RNA extraction was performed on an Iprep machine (Invitrogen) using the Iprep Pure Link Virus Kit (Invitrogen) according to the manufacturer’s instructions. RNA was eluted with buffer provided in the kit to make a final volume of 50 μL. All RNA samples were stored at < -65 °C until use. The RNA extracts were used for the real-time RT-qPCR and conventional RT-PCR followed by Sanger sequencing.

#### RNA extraction for samples subjected to high throughput sequencing (HTS)

A total of 22 samples were subjected to HTS: seven samples from Moldova and 15 from north-eastern Romania (marked with #, see Table 1). RNA was extracted from 150 μL of clarified supernatant of a 5% (1 g of brain and 4 mL of PBS) brain suspension for each of the 22 brain samples using a NucleoSpin RNA Virus kit (Macherey Nagel, Hoerdt, France), according to the manufacturer’s instructions and eluted in a final volume of 400 μL. RNA was stored at < -65 °C. RNA was depleted of host genomic DNA using the on-column DNAse digestion protocol with an RNeasy Plus mini kit (QIAGEN, Les Ulis, France), following the manufacture’s instructions.

### Real-time RT-qPCR technique

A one step SYBR-Green Real-Time RT-qPCR technique was used to detect all *Lyssavirus* species rabies virus N genes [20,21]. The technique was performed as previously described [22] using a QuantiTect SYBR Green RT-PCR kit (QIAGEN) and pan-*Lyssavirus* primers (JW12 and N165-146). The RT-PCR mixtures (final volume of 25 μL) contained 23 μL of master mix, 600 nmol of each forward and reverse primer (Table 2) and 2 μL of RNA.

**Table 2:** Characteristics of primers used in both real-time RT-qPCR and conventional RT-PCR techniques.

Gene amplified	Method	Primers	Sequence (5’ 3’)	Amplified target	PCR fragment (bp)	Sens	Reference
**Full N gene**	Conventional RT-PCR	JW12	ATG TAA CAC CYC TAC AATG	55–73	1531	Forward	[23]
		PVN8-GT1	AGT YTC TTC RGC CAT CTC	1585–1568		Reverse	This study
	Sequencing PCR	M13-JW12	GTA AAA CGA CGG CCA GA TGT AAC ACC YCT ACA ATG	55–73	1468	Forward	This study
		M13RevPVN8Bis	CAG GAA ACA GCT ATG ACC TTG CTC ATA YTT GGG	1507–1522		Reverse	This study
**Partial N gene**	Real-Time RT-qPCR	JW12	ATG TAA CAC CYC TAC AAT G	55–73	111	Forward	[21]
		N165-N146	GCA GGG TAY TTR TAC TCA TA	165–146		Reverse	

The mixtures were amplified on a Rotor-Gene Q Real-Time Cycler (QIAGEN), with the following amplification cycles: 50 °C for 30 min, 95 °C for 15 min, followed by 45 cycles at 94 °C for 30 sec, 55 °C for 30 sec and 72 °C for 30 sec. A serial dilution (from 10^7^ to 10^0^ copies RNA/μL) of an *in vitro* RABV RNA transcript at a concentration of 10^7^ copies/ μL was performed to determine PCR efficiency and the coefficient of determination (R^2^) for each assay. Negative (negative RNA extraction and PCR control) and positive (Duvenhage lyssavirus—DUVV) controls were included in each assay. The Ct values were determined according to the PCR cycle number at which the fluorescence crosses a statistically higher value than the background, which is automatically set by the software (usually set to 0.05).

### Conventional RT-PCR

All 37 RNA extracts were subjected to full-length N gene (1353 bp) amplification of the RABV. Conventional RT-PCR was performed in a final volume of 15 μL with 5 μL of RNA, 3 μL of QIAGEN One-step RT-PCR kit (QIAGEN), 0.525 μL of forward primer (20 μM), 0.525 μL of reverse primer (20 μM) (Table 2), 0.6 μL dNTP (10 mM), 0.5 μL RNAse inhibitor and 0.6 μL QIAGEN RT-PCR enzyme. Thermocycling conditions were the following: one cycle of reverse-transcription at 50 °C for 30 min, one cycle of denaturation at 95 °C for 15 min, followed by 16 cycles at 94 °C for 30 sec, a 0.4 °C touch down decrease of the annealing temperature from 57 °C to 51 °C for 30 sec and 2 min at 72 °C, then 29 cycles at 94 °C for 30 sec, at 55 °C for 30 sec, at 72 °C for 2 min and a final extension at 72 °C for 10 min.

Two μL of the first RT-PCR products were amplified in a final volume of 20 μL with 0.25 μL of both sequencing primers M13-JW12 and M13RevPVN8Bis, 1 μL of dNTP (2 mM), 1 μL of MgCl_2_ (50 mM) and 0.25 μL of Platinum Taq DNA polymerase (5 U/μL) (Invitrogen). The PCR conditions were: one cycle of denaturing at 94 °C for 2 min, followed by 45 cycles at 94 °C for 30 sec, at 48 °C for 30 sec and at 72 °C for 2 min and a final extension at 72 °C for 7 min.

PCR products were analysed by electrophoresis. The PCR products were separated on 2% agarose gel, stained with SYBR Safe solution (Invitrogen) at a final concentration of 1/10.000, visualised and photographed on a Gel Imaging system (Gel Doc XR+, Bio-Rad, Marnes-la-Coquette, France).

### SANGER sequencing

Following amplification, all 37 PCR products (full-length N gene) were SANGER sequenced by a sequencing service provider (Eurofins Genomics Cologne, Germany; Beckman Coulter Genomics, Takeley, United Kingdom and Genoscreen, Lille, France).

### High throughput sequencing (HTS)

The high throughput sequencing was performed on seven samples (all from domestic animals: goat = 1, dog = 1, cow = 3 and cat = 2) from Moldova, and 15 (five domestic animals: cat = 1, dog = 2 and cow = 2 and 10 wild animals: fox = 8, wolf = 1 and deer = 1) from north-eastern Romania (marked with #, see Table 1). HTS was undertaken at the national ANSES HTS platform (Ploufragran, France), as previously described [24]. Samples DR1019, DR1021 and DR1200 were subjected to HTS by Helixio, France.

Ion Torrent HTS methodology. For the 19 samples processed by ANSES Ploufagran, HTS was undertaken on the RNA extract after a rRNA depletion step with a Low Input Ribo Minus Kit (Ambion, Austin, TX, USA), as described by the manufacturer. cDNA libraries were prepared using an Ion Total RNA-Seq Kit (Life Technologies, Carlsbad, CA, USA) according to a protocol adapted from the supplier’s instructions (available upon request from the authors). The cDNA libraries were enriched and sequenced using the Ion Proton Sequencer and an Ion PI Chip v2 (Life Technologies). The resulting reads were cleaned with Trimmomatic 0.32 software, and then a Bowtie 2 alignment was performed on *Lyssavirus* genome references. The reads were down-sampled to fit a global coverage estimation of 80 x and were submitted to the SPAdes 3.1.1 *de novo* assembler. The *de novo* contigs were then submitted to BLAST on a local nucleotide (nt) database. For each segment, the best matches were selected for a Bowtie 2 alignment, which produces very clean and robust 5’ and 3’ ends, contrary to *de novo* assemblies of viral genomes, for which 5’ and 3’ ends are sometimes incomplete. Finally, the *de novo* assemblies and the alignment on the references were compared, and the strict identities of the *de novo* and aligned sequences were assessed [25].

Illumina HTS methodology. For the three samples DR1019, DR1021 and DR1200, RNA (112–237 ng/μL) was treated with DNAse using an RNase-Free DNase kit (QIAGEN), purified with a commercial kit RNACleanup and Concentration, then eluted in a final volume of 14 μL with RNase-free water. cDNA libraries were prepared from 1 μg of purified RNA using a TruSeq Stranded mRNA Sample Preparation kit (Illumina, San Diego, CA, USA), deleting the purification step of polyA RNA. The construction of the cDNA libraries was checked on a Bioanalyser with an Agilent DNA1000 kit and then indexed for each sample. The final products varied between 257 and 261 bp. The cDNA libraries were sequenced with a NextSeq500 Mid Output kit v2 (150 cycles) on the NextSeq 500 Sequencer (Illumina).

Bioinformatics analysis of the three samples subjected to HTS (DR1019, DR1021 and DR1200) was directly undertaken by the sequencing service provider, Helixio (France). Briefly, the resulting reads were aligned with Bwa software (http://bio-bwa.sourceforge.net/) on the GenBank reference sequence KF154997. The algorithm used was suitable for reads over 70 bp from Illumina technology. The consensus sequence was generated after cleaning the reads to keep only reads correctly appearing: both reads of a pair are aligned to the reference sequence, and the insert length is as expected. Variant calling was performed on the filtered reads using SAMtools (Tools for alignments in the SAM format, v1.3) and BCFtools (Tools for variant calling and manipulating VCFs, v1.3).

### Phylogenetic analysis

The phylogenetic analysis of the whole genome sequences was performed with a dataset of sequences constituted by 12 sequences from Romania (8 wild and 4 domestic animals) and 6 sequences from Moldova (6 domestic animals) and 37 referenced GenBank sequences (S5 Table). The dataset of sequences constituted with the samples from Romania and Moldova was cleaned to remove ambiguities (N), concatenated and trimmed using MEGA-X. After alignment of the dataset of sequences, phylogenetic analysis was assessed using SeaView software and PhyML, general time reversible (GTR) calculation and 100 bootstraps, respectively. The tree was then vizualised and annotated in FigTree 1.4.2 to include bootstrap values.

The 37 SANGER N gene consensus sequences of this study were analyzed and edited using Vector NTI software (Invitrogen, France). The dataset of sequences constituted for the phylogeny of the full length gene (*n* = 69) was then aligned and trimmed with MEGA-X. A phylogenetic tree was constructed for this analysis with 23 sequences (12 wild and 11 domestic animals) from Romania, 14 sequences (one wild and 13 domestic animals) from Moldova and 32 referenced GenBank sequences of Asian, Arctic-Related and Europe (C, D, SF, CE, WE, EE and NEE) phylogenetic groups. Phylograms was carried out using PhyML of the software Seaview with the GTR parameter and 100 boostraps.

The alignment and analysis of the amino acid (aa) and nucleotide (nt) similarities of each gene were undertaken using the Sequence Identity Matrix programs within the BioEdit software.

### Bayesian phylogenetic analysis: evolution and dates of divergence

A data set of N gene sequences was built using 37 sequences from this study (14 from Moldova and 23 from Romania) and 14 partial N gene sequences from Romania extracted from Genbank, previously published by Turcitu et al. [26]. The data set of partial N gene sequences represented the following RABV lineages: RO#1 (n = 5) and RO#2 (n = 3) clustering with the Russian D group, RO#5 (n = 4), RO#6 (n = 34) and RO#7 (n = 5) clustering with the NEE group.

The set of sequences was aligned and trimmed using MEGA V 10.1.8, resulting in a 493 bp region of N gene for BEAST analysis. The alignment of sequences was imported into BEAST V 1.8.4 to carry out Bayesian phylogenetic analysis and estimate sample dates of divergence. jModelTest [27] was used to determine the best fitting nucleotide substitution model based on the Bayesian Information Criterion (BIC) value. The analysis used the SRD06 model of nucleotide substitution (HKY85), a relaxed uncorrelated (log-normal) clock and the constant population size model. Five independent runs of 10^8 generations were conducted and sampled every 10^5 states. The log and tree files of each run were combined using LogCombiner V 1.8.4. with a burn-in of 10%. Log files were analysed in Tracer v1.7.2 to ensure that effective sample size (ESS) values were beyond the threshold (>200). The tree was obtained with TreeAnnotator V 1.8.4, then visualised and annotated on FigTree 1.4.2 with posterior probability values, time scales and age node.

## Results

### Rabies diagnosis

All the samples from the Republic of Moldova (n = 14) and north-eastern Romania (n = 23) were confirmed positive by DFA testing and cell culture tests, respectively. The same samples were also confirmed positive by real-time RT-qPCR with Ct values ranging from 13.76 to 21.68 (S1 Table). The conventional RT-PCR (full-length N gene) was shown to be positive for all 37 samples, both from Moldova and north-eastern Romania.

### Sequencing (SANGER and HTS)

Full-length N gene (1353 bp) sequences were obtained for all 37 samples through Sanger sequencing.

Concerning HTS analysis, of 22 samples selected for whole genome sequencing of the rabies virus, one sample (DR1350) produced no sequence due to degradation or insufficient quantity of the extracted RNA and was rejected from the phylogenetic study, three samples (DR1034, DR1035 and DR1187) were also excluded and other three samples (DR1036, DR1331 and DR1345) produced partial sequences. The 15 remaining samples produced full sequences of the rabies virus genome, ranging from 11,801 to 11,923 bp (S2 Table).

Although 4 samples (DR1350, DR1034, DR1035 and DR1187) were excluded from the whole genome phylogenetic study, a full-length (1353 bp) N gene sequences were obtained for all samples, which concluded that all belonged to RABV. The 15 rabies virus genomes showed high nucleotide (nt) and amino acid (aa) similarities. Nt similarities were between 97–100% for the N gene, 97–100% for the P gene, 97–100% for the M gene, 97–100% for the G gene and 97–99% for the L gene. Aa similarities were 99–100% for the N gene, 97–100% for the P gene, 98–100% for the M gene, 98–100% for the G gene and 98–100% for the L gene (S3 Table). The calculation of the aa and nt similarities for DR1034, DR1035 and DR1187 was not feasible because of the insufficient quality of the sequences and the large number of nucleotide ambiguities.

### Phylogenetic and Bayesian analysis: Evolution and time scale

A total of 37 RABV whole genomes and 32 RABV full-length N gene representative of the RABV variants circulating across the region were recovered from GenBank for the purpose of phylogenetic comparison with the study samples (S4 and S5 Tables).

**Fig 2:**
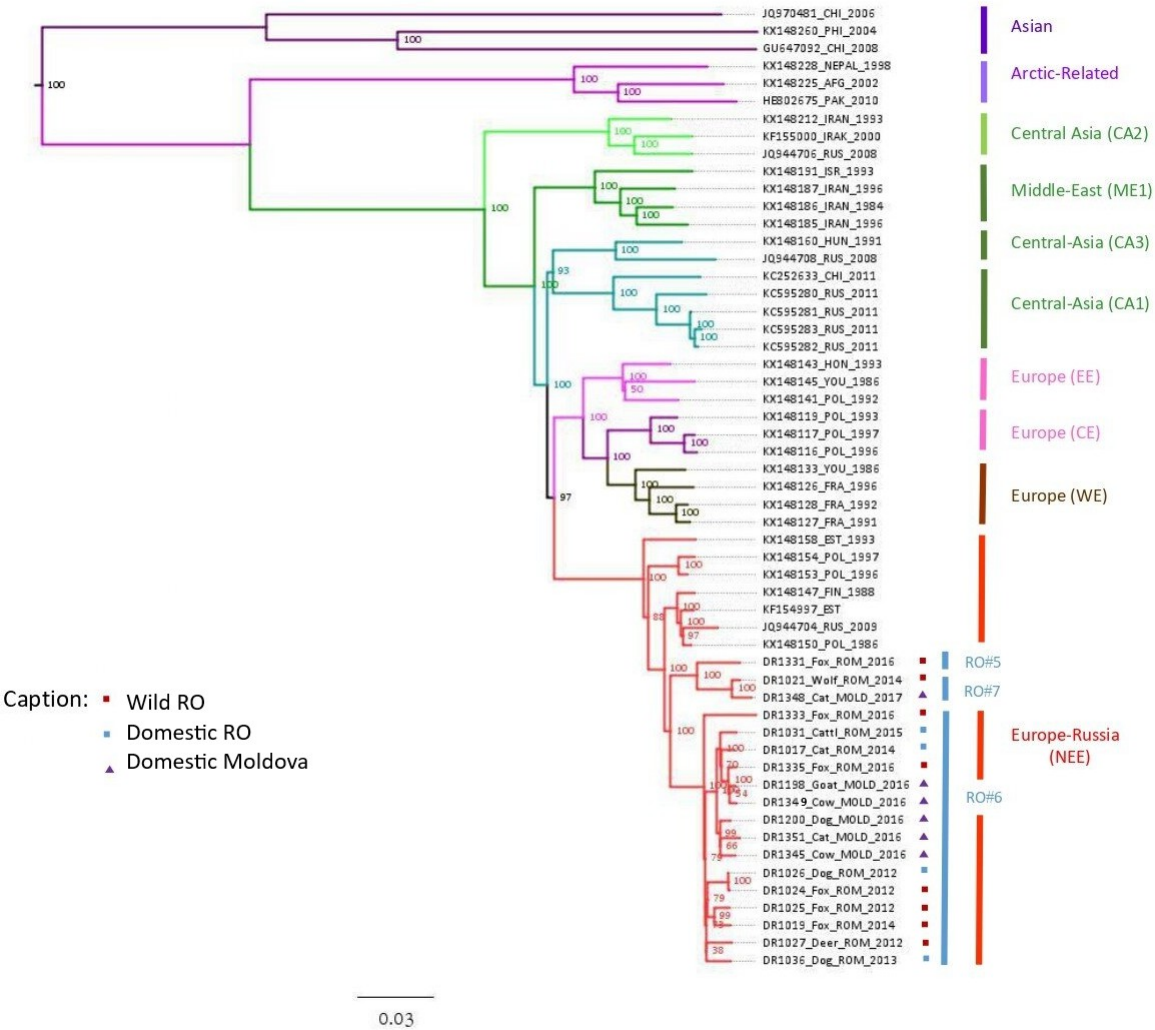
Phylogenetic tree of rabies virus whole genome for Moldova and north-eastern Romania, generated by PhyML and Seaview software. For Moldova, domestic animals (n = 6) are shown with purple triangles. For north-eastern Romania, domestic animals (n = 4) are shown with blue squares and wild animals (n = 8) with red squares.

The phylogenetic analysis performed using the PhyML and Seaview software, with GTR calculation and 100 bootstraps, is presented for the full-length N gene in S1 Fig and for the rabies virus whole genome in Fig 2.

**Fig 3:**
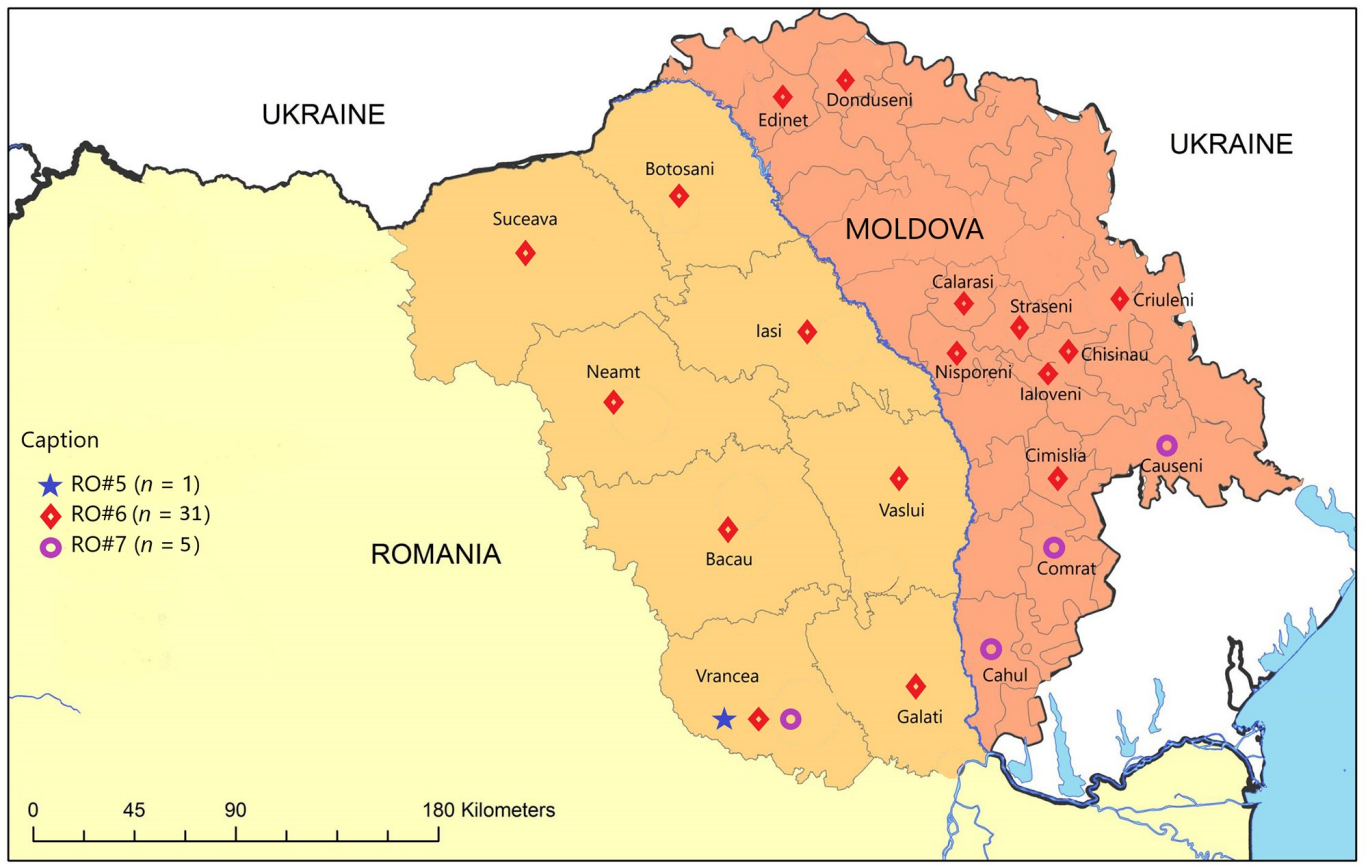
Map of Moldova and north-eastern Romania. Distribution of the three lineages RO#5, RO#6 and RO#7 in the two countries. RO#5 represented by DR1331 was isolated in a red fox, in 2016, in Vrancea county, Romania. RO#6 is present in both countries, with samples isolated between 2012 and 2016, from different animal species: red fox, cow, goat, ferret, dog, cat and deer. RO#7 included samples from both countries, isolated in 2013, 2014, 2016 and 2017, from red fox, wolf, cat and a cow. The map depicted in Fig 3 is originally and was created using authorised license of ArcGIS, version 10.5.1.

All the samples tested in our study, irrespective of the year of isolation (2012–2017) and the species, belonged to a single phylogenetic group: NEE, clustered into three assigned lineages RO#5, RO#6 and RO#7 (Fig 2). The three lineages represent distinct geographical regions and are distributed in both countries (Fig 3).

In the phylogenetic tree of the rabies virus whole genome (Fig 2), the NEE group contained different samples from Romania (n = 12), Moldova (n = 6) and representative sequences from Russia (n = 1), Poland (n = 3), Estonia (n = 2) and Finland (n = 1).

The RO#5 lineage contained only one sequence (DR1331—red fox) from Vrancea, Romania, which does not share a direct border with Moldova.

The RO#6 lineage clustered the majority of sequences: 10 from Romania (DR1333—red fox, DR1031—cow, DR1017—cat, DR1335—red fox, DR1026—dog, DR1024—red fox, DR1025—red fox, DR1019—red fox, DR1027—deer and DR1036—dog) and five from Moldova (DR1198—goat, DR1349—cow, DR1200—dog, DR1351—cat and DR1345—cow). The Romanian samples were from 7 out of 8 counties included in the present study, from which Iasi, Vaslui and Galati counties share direct borders with Moldova. For the neighbouring country, the five samples were isolated throughout the whole territory, except the south of the country.

**Fig 4:**
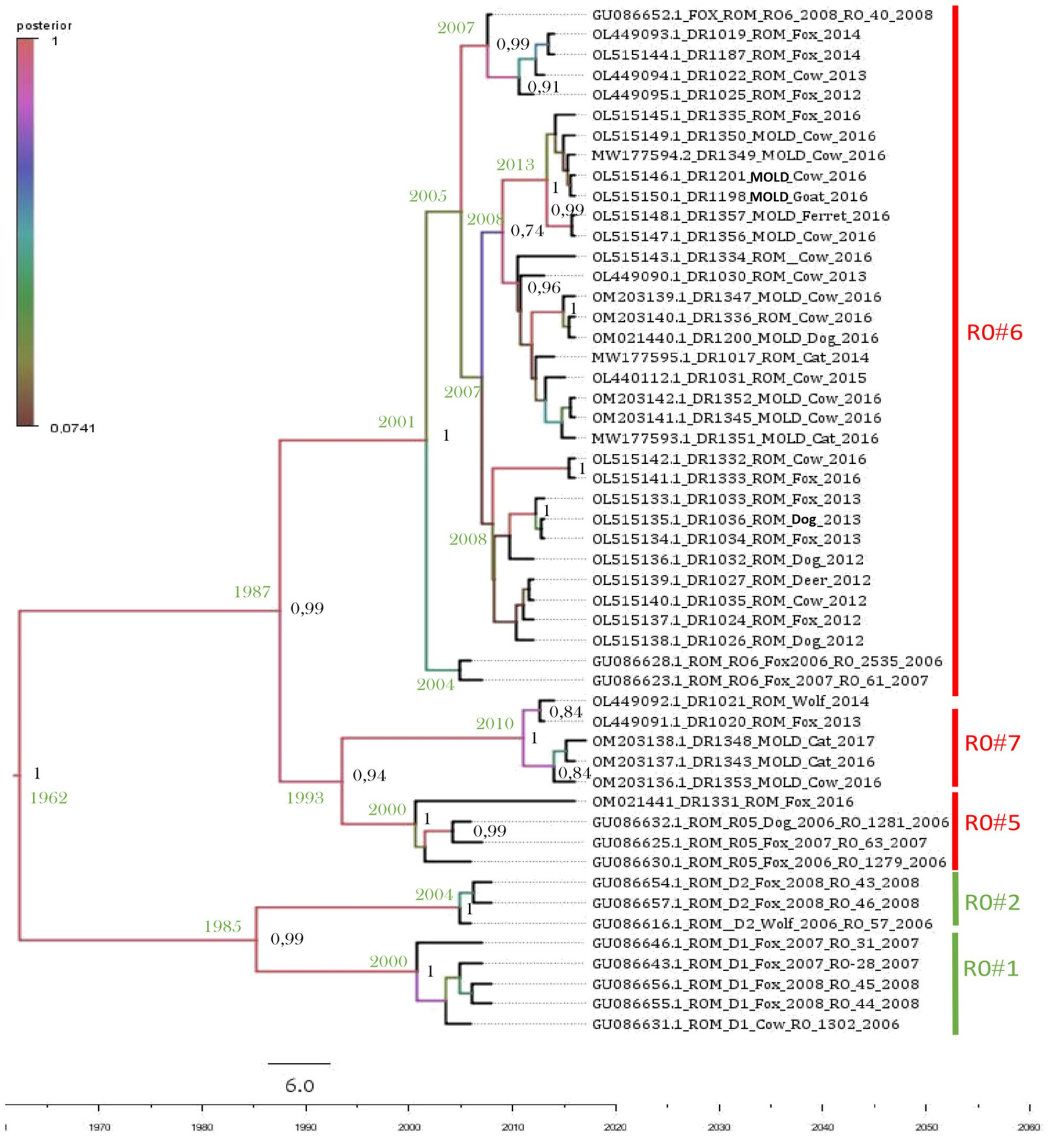
Bayesian phylogenetic analysis undertaken on partial N gene sequences from Romania and Moldova.

RO#7, a new lineage identified in the present study, included five samples, from which only two were subjected to HTS: one (DR1021—wolf) from Vrancea County, Romania and one from Comrat district, Moldova (DR1348—cat) (Fig 2). The other 3 samples were represented by DR1020—red fox, from Vrancea County Romania, DR1343—cat, from Cahul, Moldova and DR1353—cow, from Causeni, Moldova (Fig 4). None of these southern romanian and moldavian counties are sharing a direct border.

Bayesian phylogenetic analysis undertaken on the 37 partial N gene sequences from Romania and Moldova (S6 Table) revealed that all samples in the study clustered into two assigned lineages from Romania: RO#5 (1 sequence) and RO#6 (31 sequences) previously determined by Turcitu et al. [26], and a more recent one that we named RO#7 (5 sequences) (Fig 4). While RO#5 contained exclusively samples from Romania, the other two lineages contained both Moldavian and Romanian samples.

The three lineages RO#5, RO#6 and RO#7 appeared to diversify in the late 1990s early 2000s with RO#5 in ~ 2000 (95% HPD 1988–2022), RO#6 in ~ 2001 (95% HPD 1990–2022) and the more recent RO#7 in ~ 2010 (95% HPD 2006–2019).

RO#7 is formed by two wild animal samples (red fox and wolf) from Romania and 3 domestic animal samples (two cats and one cow) from Moldova. Although rabies is present in Moldova with no possible doubt, we can question the presence of the RO#7 lineage reported in Moldova in domestic animals only.

## Discussion

The phylogenetic characterisation was carried out on full sequences of N gene (n = 37) and on whole genomes of rabies virus (n = 18) from a collection of samples isolated across Moldova and north-eastern Romania. Given the absence of data in Moldova, but also in north-eastern Romania, our study provides key insights on rabies virus variant movements for this geographic area, providing advanced knowledge on the molecular evolution of rabies in these areas.

We report here the presence of the north-eastern Europe (NEE) group, clustered into three assigned lineages (RO#5, RO#6 and RO#7), in Moldova and north-eastern Romania. Both domestic and wildlife animal samples from the study area fell into the NEE group. For Moldova, the samples were isolated between 2016 (one sample coming from a wild animal and 12 from domestic animals) and 2017 (a cat sample). Concerning north-eastern Romania, the samples were isolated between 2012 and 2016, with a similar proportion between domestic and wild animals. Across Europe, at least seven phylogenetic groups of the RABV have been described since 1999, namely: C, CE, D, EE, NEE, SF and WE [3]. These different groups have been shown to further subdivide into multiple clades, each maintained by a particular host reservoir occupying a specific geographical range [6].

To the authors’ best knowledge, these are the first data on molecular characterisation of rabies virus strains from domestic and wild animals ever reported on the territory of Moldova. In Romania, a single study on the molecular characterisation of rabies virus strains was previously published in 2010 [26], with samples isolated from both domestic (cow = 3, dog = 6, cat = 7, sheep = 1, horse = 1) and wild (red fox = 41, wolf = 3, jackal = 1) animals, over a 3-year period (from 2005 to 2008) from a large part of the territory. However, the north-east of the country was less widely investigated, where only 3 (Suceava, Neamt and Bacau) out of 8 counties included in our study were covered and reported with NEE variants [26]. This NEE group was reported here in Botosani, Iasi, Vaslui, Galati and Vrancea counties of north-eastern Romania.

In the study of Turcitu et al. [26], molecular characterisation was limited to partial N gene sequencing, and six different lineages (RO#1 to RO#6), clustering into NEE, D and EE RABV variants, were identified. Besides the NEE group identified in the present study in north-eastern Romania, two other variants (D and EE groups) were also reported in the study of Turcitu et al. [26]. Moreover, group D was found in 14 counties, located mainly in the centre and south of Romania, an area limited by the Carpathian Mountains. This natural barrier limits the spread of the rabies virus, which could explain the presence of this group only in this part of the country. The EE group was found only in one case in a sample from Bihor county, located in the west [26]. The EE group reported in a single county from western Romania [26] was identified over the years in different countries such as Hungary, Poland, Serbia, Slovenia, Bosnia and Herzegovina, Czech Republic, Montenegro, Macedonia, Croatia, Bulgaria, Romania, Austria and Turkey [2,3,26,28,29].

Lineages RO#5 and RO#6 from the study of Turcitu et al. [26] were also identified in the present study. In the study of Tucitu et al. [26] lineage RO#5 was formed by 14 isolates from the south-east of the country (Tulcea, Braila, Ialomita, Constanta and Harghita) which are located outside our study area. Lineage RO#6 of Turcitu et al. [26] included 15 isolates originating from the north-west (Satu Mare), north-east (Suceava, Bacau, Neamt) and centre of the country (Valcea).

A novel finding in this study was the discovery of a new lineage RO#7 identified in five samples, two from Romania (Vrancea county) and three from Moldova (Cahul, Comrat and Causeni districts). The new lineage identified in the south of Moldova and south-east of Romania could be a result of animal movements coming from Ukraine, where the incidence of animal rabies is high. Another possibility for the new lineage could be an extension of a rabies area from Poland [30], a country that shares a border with Ukraine.

The NEE group has a broad geographic distribution, and it has been identified over the years in various countries such as Poland, Estonia, Lithuania, Latvia, Finland, Bulgaria, Slovakia (eastern region), Ukraine, Romania and Russia [2,3,10,26,28,29,31–34]. In these countries, the isolates originated mainly from foxes and raccoons, highlighting that both species are reservoirs for this virus variant. In a few cases, the NEE group was identified in badgers [3,34], marten [10], polecats [10], and one rat [3], but also in domestic animals, such as dogs [3,10], cats [2,3,10,26,28] and cattle [26,34].

In this study, the samples from north-eastern Romania originated similarly from wild and domestic animals (12 vs 11), while those from Moldova originated mainly from domestic animals (13 vs 14), demonstrating that this variant can be transmitted to many wild and domestic animal species. Considering that the samples included in the study were from different species, origins and years of collection, the Ct values obtained in molecular techniques varied from 13.76 to 21.68, suggesting that some of the samples were more or less infected by the rabies virus. Our study showed a high nucleotide and amino acid similarity (97–100%) between isolates, suggesting a possible recent spread of RABV and its maintenance in the area, by cross-species transmission across the study area.

The administrative borders of the countries do not stop the movements of different rabies virus variants. Since both Moldova and Romania are still infected and surrounded by neighbouring countries where rabies is still evolving, particularly in Ukraine, tracking the circulation and distribution of RABV variants is essential.

The entire border between Moldova and Romania is exclusively fluvial (Prut river with a length of 681.3 km). Although a river can be considered a natural barrier, limiting the circulation of infected animals from one region to another and disease distribution, it is not an absolute one, as has been demonstrated previously [3,28,35]. Such boundaries cannot stop animal movements through bridges, dams or other human infrastructures and also during winter, when the water freezes, due to the low temperatures recorded in this part of Europe. This leads to the spread of the disease and implicitly of rabies virus variants from one geographic area to another [10].

Although Romania has been considered one of the European countries heavily affected by animal rabies [15], the implementation of a strategic programme regarding the surveillance and control of rabies in foxes for a 10-year period led to a substantial decrease in positive cases that may explain the presence of only one variant. The first year of ORV campaign implementation was 2011 and the bait distributions have not been implemented systematically twice a year as recommended [36]. ORV also included the bordering areas with Hungary, Serbia and a part of Ukraine. From 2018 to 2020, a new programme was approved by the EU [36], covering all the country and including 100 km of buffer areas from 6 regions of Ukraine and 50 km radius of buffer zones from the border of Romania, covering 25 counties of Moldova. Twenty-eight animal rabies cases were reported in 2022 [37].

Both Romania and Moldova share borders with Ukraine. Two phylogenetic groups have been identified in Ukraine so far: C and NEE [10]. The C group was mainly isolated in the eastern part of the country, beyond the Dnieper River, considered a natural barrier for limiting the circulation of rabies. In contrast, the NEE group has a broader distribution area and specifically around Moldova. This may explain the presence of this variant in this part of the region.

The current context in Ukraine, with local population unfortunately heavily affected by war, has led to displacement of many refugees together with their pets to Romania. Given the unplanned departure of people from Ukraine, most of the pets that entered Romania were of unknown and/or non-compliant health status concerning rabies vaccination, putting the animal population at risk.

Romania borders Bulgaria to the south, mainly separated by a natural barrier, the Danube River. Little information is available regarding the molecular characterisation of the rabies virus in Bulgaria. Since the first description of rabies isolates, suggesting the presence of a specific Bulgarian group but also possible existence of a sub-lineage between samples collected from eastern and western districts [38], other studies revealed the presence of the EE and SF groups [2], respectively D, EE and NEE groups [28] on the territory of Bulgaria. Since the D and NEE groups were also identified in Romania [26], their presence in the north-western part of Bulgaria may be explained by the movement of infected animals that could come either from Romania or from those that transit from Ukraine or Moldova [28].

Concerning Serbia, a study [2] on the phylogenetic analysis of 175 rabies isolates collected between 1970 and 2006 revealed the presence of two groups: EE (identified in northern Serbia) and SF (with most isolates from the north and central part of the country). Identifying the EE group in Romania [26] provides evidence for trans-border movements of rabies virus variants between countries.

In Hungary, phylogenetic analysis has shown that five studied isolates clustered into the EE variant of rabies virus [3], while a recent study reported the NEE variant [39]. The EE group has also been identified [26] in one sample from Bihor county, located in the west of Romania, close to the border with Hungary, which may represent evidence for trans-border movements of rabies virus variants.

As rabies in wildlife does not respect borders, besides identifying the presence of rabies in a country, valuable information can be obtained through sequencing to characterise the circulating variants and determine which host reservoir species are responsible for virus maintenance and geographic distribution, but also to provide critical information regarding control and vaccination strategies [40].

Over the years, the rabies virus has demonstrated wide geographic and species adaptability. Rabies elimination in both Moldova and Romania will require sustained cooperation with neighbouring countries, including Ukraine, Bulgaria, Serbia and Hungary, to establish and maintain buffer zones of ORV to build an immunity belt in the target species. High throughput sequencing of samples from both domestic and wild animals was performed for the first time for both countries included in our study. These data provide new insights into RABV evolution and epidemiology in these understudied areas and expand our understanding of this disease.

## Conclusions

Phylogenetic analysis of the full-length N gene and whole genome sequences of rabies virus showed that all the samples tested in our study, from Moldova and north-eastern Romania, regardless of the year of isolation (between 2012 to 2017) and the species, belonged to a single phylogenetic group: NEE, clustering into three assigned lineages RO#5, RO#6 and RO#7.

The NEE group was identified in all tested species, namely dog, cat, cattle, fox, wolf, deer and ferret, demonstrating that this variant can be transmitted to many wild and domestic animal species.

These are the first data on molecular characterisation of rabies virus strains from domestic and wild animals reported for the territory of Moldova. Regarding north-eastern Romania, the NEE group has been identified for the first time in the following counties: Botosani, Iasi, Vaslui, Galati and Vrancea. Further investigation is needed with a larger number of samples for a more comprehensive analysis of RABV variants.

## Supporting information

S1 Table Results of real-time RT-qPCR. (DOCX)

S2 Table Results obtained for the samples subjected to high throughput sequencing (HTS). (DOCX)

S3 Table Percentage of amino acid (aa) and nucleotide (nt) similarities of Moldavian and Romanian rabies virus whole genome genes. (DOCX)

S4 Table Metadata associated with the sequences used in the molecular epidemiology study for the full-length N gene (1353 bp) of rabies virus. (DOCX)

S5 Table Metadata associated with the sequences used in the molecular epidemiology study for the whole genome of rabies virus. (DOCX)

S6 Table Metadata associated with the sequences used for the Bayesian phylogenetic analysis of the partial N gene of rabies virus. (DOCX)

S1 Fig Phylogenetic tree of rabies virus full-length N gene (1353 bp), for both Moldova and north-eastern Romania, generated by PhyML and Seaview softwares. For Moldova, domestic animals (n = 13) are shown in purple triangles and the wild animal (n = 1) in a green triangle. For north-eastern Romania, domestic animals (n = 11) are shown in blue squares and wild animals (n = 12) in red squares.
(TIFF)
