# Supplementary material for: Whole genome sequencing and phylogenetic characterisation of rabies virus strains from Moldova and north-eastern Romania
Source: PLoS Negl Trop Dis. 2023 Jul 6;17(7):e0011446. doi: 10.1371/journal.pntd.0011446 (PMC10325106; doi:10.1371/journal.pntd.0011446)
Supplement: S1 Table — (DOCX) [file pntd.0011446.s001.docx]

**S1 Table. Results of real-time RT-qPCR.**

| No. | Tested sample | SYBRGreen RT-qPCR | | | No. | Tested sample | SYBRGreen RT-qPCR | | |
| --- | --- | --- | --- | --- | --- | --- | --- | --- | --- |
|  |  | **Ct** | **< Ct** | **Standard deviation** |  |  | **Ct** | **< Ct** | **Standard deviation** |
| 1 | DR1017 | 15.30 | 15.36 | 0.09 | **23** | DR1333 | 16.76 | 16.81 | 0.08 |
|  |  | 15.42 |  |  |  |  | 16.87 |  |  |
| 2 | DR1019 | 13.82 | 13.76 | 0.08 | **24** | DR1334 | 16.19 | 16.20 | 0.00 |
|  |  | 13.71 |  |  |  |  | 16.20 |  |  |
| 3 | DR1020 | 16.13 | 16.21 | 0.12 | **25** | DR1335 | 16.10 | 16.39 | 0.41 |
|  |  | 16.30 |  |  |  |  | 16.68 |  |  |
| 4 | DR1021 | 16.00 | 15.89 | 0.15 | **26** | DR1336 | 15.98 | 15.93 | 0.08 |
|  |  | 15.78 |  |  |  |  | 15.88 |  |  |
| 5 | DR1022 | 17.19 | 17.22 | 0.04 | **27** | DR1343 | 17.08 | 17.33 | 0.35 |
|  |  | 17.25 |  |  |  |  | 17.58 |  |  |
| 6 | DR1024 | 14.72 | 14.61 | 0.16 | **28** | DR1345 | 19.03 | 19.05 | 0.02 |
|  |  | 14.49 |  |  |  |  | 19.06 |  |  |
| 7 | DR1025 | 15.72 | 15.65 | 0.10 | **29** | DR1347 | 15.75 | 15.82 | 0.10 |
|  |  | 15.57 |  |  |  |  | 15.89 |  |  |
| 8 | DR1026 | 13.89 | 13.97 | 0.12 | **30** | DR1348 | 17.66 | 17.60 | 0.09 |
|  |  | 14.06 |  |  |  |  | 17.53 |  |  |
| 9 | DR1027 | 15.17 | 15.21 | 0.06 | **31** | DR1349 | 17.72 | 17.70 | 0.03 |
|  |  | 15.25 |  |  |  |  | 17.68 |  |  |
| 10 | DR1030 | 19.57 | 19.55 | 0.03 | **32** | DR1350 | 17.33 | 17.27 | 0.08 |
|  |  | 19.52 |  |  |  |  | 17.21 |  |  |
| 11 | DR1031 | 16.03 | 16.02 | 0.01 | **33** | DR1351 | 14.03 | 14.00 | 0.04 |
|  |  | 16.01 |  |  |  |  | 13.97 |  |  |
| 12 | DR1032 | 19.57 | 19.61 | 0.06 | **34** | DR1352 | 19.24 | 19.28 | 0.06 |
|  |  | 19.65 |  |  |  |  | 19.32 |  |  |
| 13 | DR1033 | 16.31 | 16.23 | 0.07 | **35** | DR1353 | 19.25 | 19.39 | 0.20 |
|  |  | 16.38 |  |  |  |  | 19.53 |  |  |
| 14 | DR1034 | 16.15 | 16.23 | 0.11 | **36** | DR1356 | 15.57 | 15.61 | 0.06 |
|  |  | 16.30 |  |  |  |  | 15.65 |  |  |
| 15 | DR1035 | 15.80 | 15.77 | 0.04 | **37** | DR1357 | 15.36 | 15.41 | 0.06 |
|  |  | 15.73 |  |  |  |  | 15.45 |  |  |
| 16 | DR1036 | 17.21 | 17.21 | 0.01 |  | | | | |
|  |  | 17.20 |  |  |  |  |  |  |  |
| 17 | DR1187 | 23.56 | 21.68 | 0.17 |  |  |  |  |  |
|  |  | 23.64 |  |  |  |  |  |  |  |
| 18 | DR1198 | 16.92 | 15.49 | 0.05 |  |  |  |  |  |
|  |  | 16.93 |  |  |  |  |  |  |  |
| 19 | DR1200 | 21.93 | 20.91 | 0.66 |  |  |  |  |  |
|  |  | 23.41 |  |  |  |  |  |  |  |
| 20 | DR1201 | 18.01 | 16.32 | 0.02 |  |  |  |  |  |
|  |  | 17.75 |  |  |  |  |  |  |  |
| 21 | DR1331 | 15.26 | 15.28 | 0.02 |  |  |  |  |  |
|  |  | 15.30 |  |  |  |  |  |  |  |
| 22 | DR1332 | 15.78 | 15.81 | 0.04 |  |  |  |  |  |
|  |  | 15.84 |  |  |  |  |  |  |  |
