# Supplementary material for: Whole genome sequencing and phylogenetic characterisation of rabies virus strains from Moldova and north-eastern Romania
Source: PLoS Negl Trop Dis. 2023 Jul 6;17(7):e0011446. doi: 10.1371/journal.pntd.0011446 (PMC10325106; doi:10.1371/journal.pntd.0011446)
Supplement: S2 Table — (DOCX) [file pntd.0011446.s002.docx]

**S2 Table. Results obtained for the samples subjected to high throughput sequencing (HTS).**

| **No.** | **Sample code** | **QUBIT**  **(ng/µL)** | **Nanodrop (ng/µL)** | | | | **SYBR Green Real Time RT-qPCR** | | **Conventional RT-PCR** | | **Gene (pb)** | | | | | **Full genome (bp)** | **Reference sequence** |
| --- | --- | --- | --- | --- | --- | --- | --- | --- | --- | --- | --- | --- | --- | --- | --- | --- | --- |
|  |  |  | **DO260** | **DO280** | **DO260/280** | **ng/µL** | **<Ct** | ***DS** | **N606 bp** | **N1353 bp** | **N** | **P** | **M** | **G** | **L** |  |  |
| 1 | DR1017 | 53.7 | 0.719 | 0.283 | >2 | 288 | 14.22 | 0.11 | Positive | Positive | 1353 | 894 | 609 | 1575 | 6384 | 11801 | KX148147 |
| 2 | DR1024 | 49.5 | 0.531 | 0.214 | >2 | 212 | 14.05 | 0.12 | Positive | Positive | 1353 | 894 | 609 | 1575 | 6384 | 11923 | KF154997 |
| 3 | DR1025 | 74 | 0.642 | 0.262 | >2 | 257 | 13.57 | 0.08 | Positive | Positive | 1353 | 894 | 609 | 1575 | 6384 | 11910 | KC252633 |
| 4 | DR1026 | 82 | 0.65 | 0.259 | >2 | 260 | 13.05 | 0.01 | Positive | Positive | 1353 | 894 | 609 | 1575 | 6384 | 11801 | KX148150 |
| 5 | DR1027 | 26.1 | 0.446 | 0.159 | >2 | 178 | 14.37 | 0.06 | Positive | Positive | 1353 | 894 | 609 | 1575 | 6384 | 11801 | KX148150 |
| 6 | DR1031 | 28.2 | 0.516 | 0.194 | >2 | 205 | 16.71 | 0.01 | Positive | Positive | 1353 | 894 | 609 | 1575 | 6384 | 11923 | KF154997 |
| 7 | **DR1034 #2** | 31.3 | 0.773 | 0.285 | >2 | 309 | 14.7 | 0.02 | Positive | Positive | 1353 | 894 | 609 | 1575 | 6384 | 11918 | KF154997 |
| 8 | **DR1035 #2** | 37.1 | 0.52 | 0.195 | >2 | 208 | 14.86 | 0.02 | Positive | Positive | 1353 | 894 | 609 | 1575 | 6384 | 11923 | KF154997 |
| 9 | ***DR1036** | 37.7 | 0.531 | 0.188 | >2 | 212 | 16.87 | 0.11 | Positive | Positive | 1353 | 894 | NA | NA | 6384 | 11801 | KX148150 |
| 10 | **DR1187 #2** | 37.5 | 0.438 | 0.183 | >2 | 175 | 15.46 | 0.17 | Positive | Positive | 1353 | 894 | 609 | 1575 | 6384 | 11801 | KX148150 |
| 11 | DR1198 | 22.2 | 0.525 | 0.19 | >2 | 210 | 10.22 | 0.05 | Positive | Positive | 1353 | 894 | 609 | 1575 | 6384 | 11923 | KF154997 |
| 12 | ***DR1331** | 63.3 | 0.528 | 0.208 | >2 | 211 | 14.21 | 0.07 | Positive | Positive | 1353 | 894 | 609 | NA | 6384 | 11797 | KX148153 |
| 13 | DR1333 | 72.5 | 0.739 | 0.299 | >2 | 295 | 16.34 | 0.12 | Positive | Positive | 1353 | 894 | 609 | 1575 | 6384 | 11902 | KC252633 |
| 14 | DR1335 | 50.8 | 0.461 | 0.181 | >2 | 184 | 14.33 | 0.08 | Positive | Positive | 1353 | 894 | 609 | 1575 | 6384 | 11902 | KC252633 |
| 15 | ***DR1345** | 27.6 | 0.21 | 0.1 | >2 | 83 | 16.83 | 0.2 | Positive | Positive | 1353 | 894 | 609 | NA | 6384 | 11839 | KC252633 |
| 16 | DR1348 | 160 | 0.56 | 0.24 | >2 | 226 | 16.36 | 0.15 | Positive | Positive | 1353 | 894 | 609 | 1575 | 6384 | 11835 | KC252633 |
| 17 | DR1349 | 42.1 | 0.58 | 0.22 | >2 | 234 | 16.33 | 0.01 | Positive | Positive | 1353 | 894 | 609 | 1575 | 6384 | 11833 | KC252633 |
| 18 | **#DR1350** | 37.1 | 0.22 | 0.1 | >2 | 90 | 15.96 | 0.22 | Positive | Positive | **-** | **-** | **-** | **-** | **-** | **-** | **-** |
| 19 | DR1351 | 190 | 1 | 0.44 | >2 | 399 | 12.63 | 0.28 | Positive | Positive | 1353 | 894 | 609 | 1575 | 6384 | 11801 | KX148150 |
| 20 | DR1019 | 237 | 5.53 | 2.77 | >2 | 221 | 13.76 | 0.08 | Positive | Positive | 1353 | 894 | 609 | 1575 | 6384 | 11910 | KF154997 |
| 21 | DR1021 | 112 | 2.21 | 1.73 | >2 | 88 | 15.89 | 0.15 | Positive | Positive | 1353 | 894 | 609 | 1575 | 6384 | 11915 | KF154997 |
| 22 | DR1200 | 116 | 2.08 | 1.04 | >2 | 83 | 20.91 | 0.66 | Positive | Positive | 1353 | 894 | 609 | 1575 | 6384 | 11916 | KF154997 |

**#** DR1350: excluded from the analysis (incomplete sequences).

**#2** DR1034, DR1035 and DR1187: not included in the phylogenetic analysis of the whole genome sequences

***** DR1036, DR1331, DR1345: partially sequenced.
