# Supplementary material for: Whole genome sequencing and phylogenetic characterisation of rabies virus strains from Moldova and north-eastern Romania
Source: PLoS Negl Trop Dis. 2023 Jul 6;17(7):e0011446. doi: 10.1371/journal.pntd.0011446 (PMC10325106; doi:10.1371/journal.pntd.0011446)
Supplement: S3 Table — (DOCX) [file pntd.0011446.s003.docx]

**S3 Table. Percentage of amino acid (aa) and nucleotide (nt)** **similarities of Moldavian and Romanian rabies virus whole genome genes.**

| **No.** | **Sample** | **N gene** | | **G gene** | | **P gene** | | **M gene** | | **L gene** | | **Full genome (bp)** |
| --- | --- | --- | --- | --- | --- | --- | --- | --- | --- | --- | --- | --- |
|  |  | Nt  (1353nt) | AA  (450AA) | Nt  (1575nt) | AA  (524AA) | Nt  (894nt) | AA  (297AA) | Nt  (609nt) | AA  (202AA) | Nt  (6327/6384) | AA  (2091) |  |
| 1 | DR1017 | 0.984 | 0.997 | 0.98 | 0.99 | 0.978 | 0.983 | 0.977 | 0.985 | 0.984 | 0.994 | 11801 |
| 2 | DR1024 | 0.987 | 1 | 0.976 | 0.988 | 0.98 | 0.989 | 0.98 | 0.985 | 0.985 | 0.994 | 11923 |
| 3 | DR1025 | 0.983 | 1 | 0.979 | 0.992 | 0.986 | 0.986 | 0.98 | 0.985 | 0.984 | 0.995 | 11910 |
| 4 | DR1026 | 0.987 | 1 | 0.976 | 0.988 | 0.98 | 0.989 | 0.98 | 0.985 | 0.986 | 0.995 | 11801 |
| 5 | DR1027 | 0.986 | 0.997 | 0.977 | 0.994 | 0.982 | 0.989 | 0.977 | 0.98 | 0.983 | 0.993 | 11801 |
| 6 | DR1031 | 0.984 | 0.997 | 0.976 | 0.988 | 0.978 | 0.986 | 0.973 | 0.98 | 0.984 | 0.994 | 11923 |
| 7 | DR1333 | 0.982 | 0.997 | 0.976 | 0.99 | 0.975 | 0.983 | 0.975 | 0.985 | 0.979 | 0.992 | 11902 |
| 8 | DR1335 | 0.983 | 1 | 0.979 | 0.992 | 0.974 | 0.976 | 0.98 | 0.985 | 0.984 | 0.993 | 11902 |
| 9 | DR1348 | 0.977 | 0.991 | 0.977 | 0.988 | 0.978 | 0.976 | 0.973 | 0.99 | 0.98 | 0.99 | 11835 |
| 10 | DR1349 | 0.983 | 0.991 | 0.979 | 0.99 | 0.975 | 0.979 | 0.978 | 0.985 | 0.979 | 0.988 | 11833 |
| 11 | DR1351 | 0.985 | 0.997 | 0.975 | 0.992 | 0.976 | 0.983 | 0.975 | 0.98 | 0.982 | 0.994 | 11801 |
| 12 | DR1198 | 0.985 | 1 | 0.978 | 0.988 | 0.975 | 0.976 | 0.977 | 0.985 | 0.984 | 0.994 | 11923 |
| 13 | DR1019 | 0.985 | 1 | 0.98 | 0.99 | 0.982 | 0.983 | 0.98 | 0.985 | 0.983 | 0.994 | 11910 |
| 14 | DR1021 | 0.98 | 0.997 | 0.978 | 0.986 | 0.982 | 0.983 | 0.975 | 0.99 | 0.985 | 0.995 | 11915 |
| 15 | DR1200 | 0.983 | 0.997 | 0.98 | 0.99 | 0.98 | 0.986 | 0.978 | 0.985 | 0.984 | 0.994 | 11916 |

Sample DR1350 (not shown in S3 Table) excluded from the analysis (incomplete sequence).

Samples DR1036, DR1331 and DR1345 (not shown in S3 Table) were partially sequenced.

The calculation of the aa and nt similarities for DR1034, DR1035 and DR1187 (not shown in S3 Table) was not feasible because of the insufficient quality of the sequences and the large number of nucleotide ambiguities.
