# Supplementary material for: Whole genome sequencing and phylogenetic characterisation of rabies virus strains from Moldova and north-eastern Romania
Source: PLoS Negl Trop Dis. 2023 Jul 6;17(7):e0011446. doi: 10.1371/journal.pntd.0011446 (PMC10325106; doi:10.1371/journal.pntd.0011446)
Supplement: S4 Table — (DOCX) [file pntd.0011446.s004.docx]

**S4 Table. Metadata associated with the sequences used in the molecular epidemiology study for the full-length N gene (1353 bp) of rabies virus.**

| No. | Country | Region | Isolate | Species | Year of isolation | Phylogenetic group | GenBank  accession number | Reference |
| --- | --- | --- | --- | --- | --- | --- | --- | --- |
| 1 | Philippines | - | 04030PHI | Dog  *(Canis lupus familiaris)* | 2004 | Asian | KX148260 | [1] |
| 2 | China | Jiangsu Province | CJS0621D | Dog  *(Canis lupus familiaris)* | 2006 | Asian | JQ970481 | [2] |
| 3 | Nepal | - | 99001NEP | Dog  *(Canis lupus familiaris)* | 1998 | Arctic-Related | KX148228 | [1] |
| 4 | Afghanistan | - | 02052AFG | Dog  *(Canis lupus familiaris)* | 2002 | Arctic-Related | KX148225 | [1] |
| 5 | Pakistan | - | Pk 23 | Cow  *(Bos taurus)* | 2010 | Arctic-Related | HE802675 | [3] |
| 6 | Russia | Tver | Rus(Tver) 7574f_2008 | Red fox  *(Vulpes vulpes)* | 2008 | D | KJ958266 | [4] |
| 7 | Russia | Tver | Rus(Tver) 7573d_2008 | Dog  *(Canis lupus familiaris)* | 2008 | D | KJ958265 | [4] |
| 8 | Russia | - | 1564NNO | Red fox  *(Vulpes vulpes)* | 2008 | D | JQ944708 | [6] |
| 9 | Russia | Omsk | Rus(Omsk) 7444f_2008 | Red fox  *(Vulpes vulpes)* | 2008 | C | KJ958247 | [4] |
| 10 | Russia | Voronezh | Rus(Voronezh) 7513f_2008 | Red fox  *(Vulpes vulpes)* | 2008 | C | KC794010 | [5] |
| 11 | Russia | Lipetsk | Rus(Lipetsk) 8064f_2012 | Red fox  *(Vulpes vulpes)* | 2012 | C | KC538854 | [5] |
| 12 | Serbia | - | RV1154 | Fox  *(Vulpes vulpes)* | 1976 | SF | JF973776 | [7] |
| 13 | Serbia | - | RV1162 | Feline  *(Felis catus)* | 1977 | SF | JF973779 | [7] |
| 14 | Serbia | - | RV1151 | Fox  *(Vulpes vulpes)* | 1972 | SF | JF973775 | [7] |
| 15 | Poland | - | 96045POL | Fox  *(Vulpes vulpes)* | 1993 | CE | KX148119 | [1] |
| 16 | Poland | - | 97046POL | Fox  *(Vulpes vulpes)* | 1997 | CE | KX148117 | [1] |
| 17 | Poland | - | 96256POL | Fox  *(Vulpes vulpes)* | 1996 | CE | KX148116 | [1] |
| 18 | Bosnia and Herzegovina | - | 86111YOU | Fox  *(Vulpes vulpes)* | 1986 | WE | KX148133 | [1] |
| 19 | France | - | 9223FRA | Fox  *(Vulpes vulpes)* | 1974 | WE | U43433 | [8] |
| 20 | France | - | 8663FRA | Fox  *(Vulpes vulpes)* | 1984 | WE | U42605 | [8] |
| 21 | Bosnia and Herzegovina | - | 86054YOU | Wolf  *(Canis lupus)* | 1986 | EE | KX148145 | [1] |
| 22 | Montenegro | - | RV1268 | Horse  (Equus caballus) | 2000 | EE | JF973802 | [7] |
| 23 | Hungary | - | 93080HON | Fox  *(Vulpes vulpes)* | 1993 | EE | KX148143 | [1] |
| 24 | Macedonia | - | DR400 | Red fox  *(Vulpes vulpes)* | 2011 | EE | JQ973880 | [9] |
| 25 | Poland | - | 96135POL | Fox  *(Vulpes vulpes)* | 1992 | EE | KX148141 | [1] |
| 26 | Hungary | - | 93091HON | Fox  *(Vulpes vulpes)* | 1993 | EE | KX148138 | [1] |
| 27 | Serbia | - | RV1290 | Fox  *(Vulpes vulpes)* | 1999 | EE | JF973807 | [7] |
| 28 | Estonia | - | 9339EST | Raccoon dog  (*Nyctereutes procyonoides*) | 1991 | NEE | U42707 | [8] |
| 29 | Estonia | - | 9342EST | Raccoon dog  (*Nyctereutes procyonoides*) | 1991 | NEE | U43432 | [8] |
| 30 | Poland | - | 96097POL | Raccoon dog  (*Nyctereutes procyonoides*) | 1996 | NEE | KX148153 | [1] |
| 31 | Romania | Vrancea | DR1331 | Red fox  *(Vulpes vulpes)* | 2016 | NEE | OM021441 | This study |
| 32 | Romania | Vrancea | DR1020 | Red fox  *(Vulpes vulpes)* | 2013 | NEE | OL449091 | This study |
| 33 | Romania | Vrancea | DR1021 | Wolf  *(Canis lupus)* | 2014 | NEE | OL449092 | This study |
| 34 | Moldova | Causeni | DR1353 | Cow  *(Bos taurus)* | 2016 | NEE | OM203136 | This study |
| 35 | Moldova | Cahul | DR1343 | Cat  *(Felis catus)* | 2016 | NEE | OM203137 | This study |
| 36 | Moldova | Comrat | DR1348 | Cat  *(Felis catus)* | 2017 | NEE | OM203138 | This study |
| 37 | Russia | - | 184VNO | Raccoon dog  (*Nyctereutes procyonoides*) | 2009 | NEE | JQ944704 | [6] |
| 38 | Russia | Pskov | RV1596 | Fox  *(Vulpes vulpes)* | - | NEE | AY352474 | [10] |
| 39 | Romania | Suceava | DR1333 | Red fox  *(Vulpes vulpes)* | 2016 | NEE | OL515141 | This study |
| 40 | Romania | Suceava | DR1332 | Cow  *(Bos taurus)* | 2016 | NEE | OL515142 | This study |
| 41 | Romania | Vrancea | DR1022 | Cow  *(Bos taurus)* | 2013 | NEE | OL449094 | This study |
| 42 | Romania | Vrancea | DR1019 | Red fox  *(Vulpes vulpes)* | 2014 | NEE | OL449093 | This study |
| 43 | Romania | Vaslui | DR1187 | Red fox  *(Vulpes vulpes)* | 2014 | NEE | OL515144 | This study |
| 44 | Romania | Bacau | DR1025 | Red fox  *(Vulpes vulpes)* | 2012 | NEE | OL449095 | This study |
| 45 | Romania | Bacau | DR1026 | Dog  *(Canis lupus familiaris)* | 2012 | NEE | OL515138 | This study |
| 46 | Romania | Bacau | DR1024 | Red fox  *(Vulpes vulpes)* | 2012 | NEE | OL515137 | This study |
| 47 | Romania | Neamt | DR1032 | Dog  *(Canis lupus familiaris)* | 2012 | NEE | OL515136 | This study |
| 48 | Romania | Neamt | DR1035 | Cow  *(Bos taurus)* | 2012 | NEE | OL515140 | This study |
| 49 | Romania | Bacau | DR1027 | Roe deer  *(Capreolus capreolus)* | 2012 | NEE | OL515139 | This study |
| 50 | Romania | Neamt | DR1036 | Dog  *(Canis lupus familiaris)* | 2013 | NEE | OL515135 | This study |
| 51 | Romania | Neamt | DR1034 | Red fox  *(Vulpes vulpes)* | 2013 | NEE | OL515134 | This study |
| 52 | Romania | Neamt | DR1033 | Red fox  *(Vulpes vulpes)* | 2013 | NEE | OL515133 | This study |
| 53 | Moldova | Edineti | DR1347 | Cow  *(Bos taurus)* | 2016 | NEE | OM203139 | This study |
| 54 | Moldova | Criuleni | DR1200 | Dog  *(Canis lupus familiaris)* | 2016 | NEE | OM021440 | This study |
| 55 | Romania | Botosani | DR1336 | Cow  *(Bos taurus)* | 2016 | NEE | OM203140 | This study |
| 56 | Romania | Botosani | DR1334 | Cow  *(Bos taurus)* | 2016 | NEE | OL515143 | This study |
| 57 | Moldova | Dondiuseni | DR1351 | Cat  *(Felis catus)* | 2016 | NEE | MW177593 | This study |
| 58 | Moldova | Criuleni | DR1345 | Cow  *(Bos taurus)* | 2016 | NEE | OM203141 | This study |
| 59 | Moldova | Criuleni | DR1352 | Cow  *(Bos taurus)* | 2016 | NEE | OM203142 | This study |
| 60 | Romania | Iasi | DR1030 | Cow  *(Bos taurus)* | 2013 | NEE | OL449090 | This study |
| 61 | Romania | Galati | DR1031 | Cow  *(Bos taurus)* | 2015 | NEE | OL440112 | This study |
| 62 | Romania | Vaslui | DR1017 | Cat  *(Felis catus)* | 2014 | NEE | MW177595 | This study |
| 63 | Moldova | Nisporeni | DR1349 | Cow  *(Bos taurus)* | 2016 | NEE | MW177594 | This study |
| 64 | Moldova | Straseni | DR1356 | Cow  *(Bos taurus)* | 2016 | NEE | OL515147 | This study |
| 65 | Moldova | Ialoveni | DR1357 | Ferret  *(Mustela putorius furo)* | 2016 | NEE | OL515148 | This study |
| 66 | Moldova | Chisinau | DR1198 | Goat  *(Capra aegagrus hircus)* | 2016 | NEE | OL515150 | This study |
| 67 | Moldova | Cimislia | DR1350 | Cow  *(Bos taurus)* | 2016 | NEE | OL515149 | This study |
| 68 | Romania | Iasi | DR1335 | Red fox  *(Vulpes vulpes)* | 2016 | NEE | OL515145 | This study |
| 69 | Moldova | Calarasi | DR1201 | Cow  *(Bos taurus)* | 2016 | NEE | OL515146 | This study |

* D: Center of the European part of Russia; C: European part of Russia; SF: Serbian fox; CE: Central Europe; WE: Western Europe; EE: Eastern Europe; NEE: North-Eastern Europe.

**REFERENCES**

1. Troupin C, Dacheux L, Tanguy M, Sabeta C, Blanc H, Bouchier C, et al. Large-Scale Phylogenomic Analysis Reveals the Complex Evolutionary History of Rabies Virus in Multiple Carnivore Hosts. PLoS Pathog. 2016;12(12):1–20.
2. Tang Q, Zhang J, Li H, Sheng XX and Liang GD. Molecular characterization of the complete genome of rabies virus isolated in China. GenBank Sequence Accession number: JQ970481 (submitted on 24-APR-2012).
3. Hussain Z, Haider MS, Qureshi ZU, Afzaal S, Villa AV, Xiangfu W and Rupprecht CE. Molecular characterization of Pakistani strains of Rabies virus. GenBank Sequence Accession number: HE802675 (submitted on 17-APR-2012).
4. Poleshchuk EM, Tkachev SE, Sidorov GN, Saryglar LK, Mangush RM, Shmatova LV, Demchin PM, Lutsenko GV and Glushkov VV. The rabies of wild mammals from the south Eastern Siberia, Russia in the beginning of the XXI century. GenBank Sequence Accession numbers: KJ958266, KJ958265 and KJ958247 (submitted on 05-JUN-2014).
5. Poleshchuk EM, Sidorov GN, Tkachev SE, Devyatkin AA, Dedkov VG, Ochkasova JV, Hodjakova IA, Schukina IA, Savelev SI and Golenskih AG. Rabies in the center of the East European Plain (Lipetsk region, Russia. GenBank Sequence Accession numbers: KC794010 (submitted on 15-MAR-2013) and KC538854 (submitted on 28-JAN-2013).
6. Chupin S, Chernyshova E and Metlin A. Complete genome analysis of five rabies virus isolates from Russia. GenBank Sequence Accession numbers: JQ944708 and JQ944704 (submitted on 17-APR-2012).
7. McElhinney LM, Marston DA, Freuling CM, Cragg W, Stankov S, Lalosević D, et al. Molecular diversity and evolutionary history of rabies virus strains circulating in the Balkans. J Gen Virol. 2011;92(9):2171–80.
8. Bourhy H, Kissi B, Kulonen K, Tordo N, Audry L and Stohr K. Evolution of the nucleoprotein gene and host range of old world rabies virus. GenBank Sequence Accession numbers: U43433 and U43432 (submitted on 18-DEC-1995), U42605 (submitted on 08-DEC-1995) and U42707 (submitted on 11-DEC-1995).
9. Picard-Meyer E, Mrenoshki S, Milicevic V, Ilieva D,Cvetkovikj I, Cvetkovikj A et al. Molecular characterisation of rabies virus strains in the Republic of Macedonia. Arch Virol. 2013 Jan;158(1):237-40. doi: 10.1007/s00705-012-1466-9.
10. Kuzmin I V., Botvinkin AD, McElhinney LM, Smith JS, Orciari LA, Hughes GJ, et al. Molecular epidemiology of terrestrial rabies in the former Soviet Union. J Wildl Dis. 2004;40(4):617–31.
