# Supplementary material for: Whole genome sequencing and phylogenetic characterisation of rabies virus strains from Moldova and north-eastern Romania
Source: PLoS Negl Trop Dis. 2023 Jul 6;17(7):e0011446. doi: 10.1371/journal.pntd.0011446 (PMC10325106; doi:10.1371/journal.pntd.0011446)
Supplement: S5 Table — (DOCX) [file pntd.0011446.s005.docx]

**S5 Table. Metadata associated with the sequences used in the molecular epidemiology study for the whole genome of rabies virus.**

| No. | Country | Region | Isolate | Species | Year of isolation | Phylogenetic  group | GenBank accession number | Reference |
| --- | --- | --- | --- | --- | --- | --- | --- | --- |
| 1 | China | Jiangsu | CJS0621D | Dog  *(Canis lupus familiaris)* | 2006 | Asian | JQ970481 | [1] |
| 2 | Philippines | - | 04030PHI | Dog  *(Canis lupus familiaris)* | 2004 | Asian | KX148260 | [2] |
| 3 | China | - | JX08-45 | Chinese ferret badger  *(Melogale moschata)* | 2008 | Asian | GU647092 | [3] |
| 4 | Nepal | - | 99001NEP | Dog  *(Canis lupus familiaris)* | 1998 | Arctic-Related | KX148228 | [2] |
| 5 | Afghanistan | - | 02052AFG | Dog  *(Canis lupus familiaris)* | 2002 | Arctic-Related | KX148225 | [2] |
| 6 | Pakistan | - | Pk 23 | Cow  *(Bos taurus)* | 2010 | Arctic-Related | HE802675 | [4] |
| 7 | Iran | - | 93019IRA | Jackal  *(Canis aureus)* | 1993 | Central Asia (CA2) | KX148212 | [2] |
| 8 | Irak | - | RV2516 | Cow  *(Bos taurus)* | 2010 | Central Asia (CA2) | KF155000 | [5] |
| 9 | Russia | - | 1352KRA | Dog  *(Canis lupus familiaris)* | 2008 | Central Asia (CA2) | JQ944706 | [6] |
| 10 | Israel | - | 93032ISR | Jackal  *(Canis aureus)* | 1993 | Middle-East (ME1) | KX148191 | [2] |
| 11 | Iran | - | 96320IRA | Jackal  *(Canis aureus)* | 1996 | Middle-East (ME1) | KX148187 | [2] |
| 12 | Iran | - | 87002IRA | Wolf  *(Canis lupus)* | 1984 | Middle-East (ME1) | KX148186 | [2] |
| 13 | Iran | - | 96299IRA | Wolf  *(Canis lupus)* | 1996 | Middle-East (ME1) | KX148185 | [2] |
| 14 | Hungary | - | 92015HON | Human  *(Homo sapiens)* | 1991 | Central-Asia (CA3) | KX148160 | [2] |
| 15 | Russia | - | 1564NNO | Red fox  *(Vulpes vulpes)* | 2008 | Central-Asia (CA3) | JQ944708 | [6] |
| 16 | China | - | CNM1103C | Bovine  *(Bos taurus)* | 2011 | Central-Asia (CA1) | KC252633 | [7] |
| 17 | Russia | Lipetsk | Rus(Lipetsk)8052f_2011 | Red fox  *(Vulpes vulpes)* | 2011 | Central-Asia (CA1) | KC595280 | [8] |
| 18 | Russia | Lipetsk | Rus(Lipetsk)8053c_2011 | Cat  *(Felis catus)* | 2011 | Central-Asia (CA1) | KC595281 | [8] |
| 19 | Russia | Lipetsk | Rus(Lipetsk)8057f_2011 | Red fox  *(Vulpes vulpes)* | 2011 | Central-Asia (CA1) | KC595283 | [8] |
| 20 | Russia | Lipetsk | Rus(Lipetsk)8054f_2011 | Red fox  *(Vulpes vulpes)* | 2011 | Central-Asia (CA1) | KC595282 | [8] |
| 21 | Hungary | - | 93080HON | Fox  *(Vulpes vulpes)* | 1993 | EE | KX148143 | [2] |
| 22 | Bosnia and Herzegovina | - | 86054YOU | Wolf  *(Canis lupus)* | 1986 | EE | KX148145 | [2] |
| 23 | Poland | - | 96135POL | Fox  *(Vulpes vulpes)* | 1992 | EE | KX148141 | [2] |
| 24 | Poland | - | 96045POL | Fox  *(Vulpes vulpes)* | 1993 | CE | KC148119 | [2] |
| 25 | Poland | - | 97046POL | Fox  *(Vulpes vulpes)* | 1997 | CE | KX148117 | [2] |
| 26 | Poland | - | 96256POL | Fox  *(Vulpes vulpes)* | 1996 | CE | KX148116 | [2] |
| 27 | Bosnia and Herzegovina | - | 86111YOU | Fox  *(Vulpes vulpes)* | 1986 | WE | KX148133 | [2] |
| 28 | France | - | 96002FRA | Fox  *(Vulpes vulpes)* | 1996 | WE | KX148126 | [2] |
| 29 | France | - | 92044FRA | Fox  *(Vulpes vulpes)* | 1992 | WE | KX148128 | [2] |
| 30 | France | - | 91047FRA | Fox  *(Vulpes vulpes)* | 1991 | WE | KX148127 | [2] |
| 31 | Estonia | - | 93105EST | Fox  *(Vulpes vulpes)* | 1993 | NEE | KX148158 | [2] |
| 32 | Poland | - | 97078POL | Raccoon dog  *(Nyctereutes procyonoides)* | 1997 | NEE | KX148154 | [2] |
| 33 | Poland | - | 96097POL | Raccoon dog  *(Nyctereutes procyonoides)* | 1996 | NEE | KX148153 | [2] |
| 34 | Finland | - | 93048FIN | Raccoon dog  *(Nyctereutes procyonoides)* | 1988 | NEE | KX148147 | [2] |
| 35 | Estonia | - | RV437 | Raccoon dog  *(Nyctereutes procyonoides)* | - | NEE | KF154997 | [5] |
| 36 | Russia | - | 184VNO | Raccoon dog  *(Nyctereutes procyonoides)* | 2009 | NEE | JQ944704 | [6] |
| 37 | Poland | - | 96026POL | Raccoon dog  *(Nyctereutes procyonoides)* | 1986 | NEE | KX148150 | [2] |
| 38 | Romania | Vrancea | DR1331 | Red fox  *(Vulpes vulpes)* | 2016 | NEE | OM021441 | This study |
| 39 | Romania | Vrancea | DR1021 | Wolf  *(Canis lupus)* | 2014 | NEE | OL449092 | This study |
| 40 | Moldova | Comrat | DR1348 | Cat  *(Felis catus)* | 2017 | NEE | OM203138 | This study |
| 41 | Romania | Suceava | DR1333 | Red fox  *(Vulpes vulpes)* | 2016 | NEE | OL515141 | This study |
| 42 | Romania | Galati | DR1031 | Cow  *(Bos taurus)* | 2015 | NEE | OL440112 | This study |
| 43 | Romania | Barlad | DR1017 | Cat  *(Felis catus)* | 2014 | NEE | MW177595 | This study |
| 44 | Romania | Iasi | DR1335 | Red fox  *(Vulpes vulpes)* | 2016 | NEE | OL515145 | This study |
| 45 | Moldova | Chisinau | DR1198 | Goat  *(Capra aegagrus hircus)* | 2016 | NEE | OL515150 | This study |
| 46 | Moldova | Nisporeni | DR1349 | Cow  *(Bos taurus)* | 2016 | NEE | MW177594 | This study |
| 47 | Moldova | Criuleni | DR1200 | Dog  *(Canis lupus familiaris)* | 2016 | NEE | OM021440 | This study |
| 48 | Moldova | Dondiuseni | DR1351 | Cat  *(Felis catus)* | 2016 | NEE | MW177593 | This study |
| 49 | Moldova | Criuleni | DR1345 | Cow  *(Bos taurus)* | 2016 | NEE | OM203141 | This study |
| 50 | Romania | Bacau | DR1026 | Dog  *(Canis lupus familiaris)* | 2012 | NEE | OL515138 | This study |
| 51 | Romania | Bacau | DR1024 | Red fox  *(Vulpes vulpes)* | 2012 | NEE | OL515137 | This study |
| 52 | Romania | Bacau | DR1025 | Red fox  *(Vulpes vulpes)* | 2012 | NEE | OL449095 | This study |
| 53 | Romania | Vrancea | DR1019 | Red fox  *(Vulpes vulpes)* | 2014 | NEE | OL449093 | This study |
| 54 | Romania | Bacau | DR1027 | Deer  *(Capreolus capreolus)* | 2012 | NEE | OL515139 | This study |
| 55 | Romania | Neamt | DR1036 | Red fox  *(Vulpes vulpes)* | 2013 | NEE | OL515135 | This study |

* EE: Eastern Europe, CE: Central Europe, WE: Western Europe, NEE: North-Eastern Europe, ME1: Middle-East, CA1 / CA2 / CA3: Central Asia.

**REFERENCES**

1. Tang Q, Zhang J, Li H, Sheng XX and Liang GD. Molecular characterization of the complete genome of rabies virus isolated in China. GenBank Sequence Accession number: JQ970481 (submitted on 24-APR-2012).
2. Troupin C, Dacheux L, Tanguy M, Sabeta C, Blanc H, Bouchier C, et al. Large-Scale Phylogenomic Analysis Reveals the Complex Evolutionary History of Rabies Virus in Multiple Carnivore Hosts. PLoS Pathog. 2016;12(12):1–20.
3. Zhang S, Liu Y, Zhao J, Zhang F, Wang Y, Pan T and Hu R.Sequencing and Analysis of a Rabies Virus Isolate from the Chinese Ferret Badger. GenBank Sequence Accession number: GU647092 (submitted on 04-FEB-2010).
4. Hussain Z, Haider MS, Qureshi ZU, Afzaal S, Villa AV, Xiangfu W and Rupprecht CE. Molecular characterization of Pakistani strains of Rabies virus. GenBank Sequence Accession number: HE802675 (submitted on 17-APR-2012).
5. Marston DA, McElhinney LM, Ellis RJ, Horton DL, Wise EL, Leech SL et al. Next generation sequencing of viral RNA genomes. BMC Genomics. 2013 Jul 4;14:444. doi: 10.1186/1471-2164-14-444.
6. Chupin S, Chernyshova E and Metlin A. Complete genome analysis of five rabies virus isolates from Russia. GenBank Sequence Accession numbers: JQ944706, JQ944708 and JQ944704 (submitted on 17-APR-2012).
7. Yin J, Guo Z, Tao X, Tang Q, Wang F and Liang G. Molecular characterization of the complete genome of an emerging street rabies virus isolated in Inner Mongolia of China. GenBank Sequence Accession number: KC252633 (submitted on 02-DEC-2012).
8. Poleshchuk EM, Deviatkin AA, Dedkov VG, Sidorov GN, Ochkasova JV, Hodjakova IA et al. Complete genome sequences of four virulent rabies virus strains isolated from rabid animals in Russia. Genome Announc. 2013 May 9;1(3):e00140-13. doi: 10.1128/genomeA.00140-13.
