# Supplementary material for: Whole genome sequencing and phylogenetic characterisation of rabies virus strains from Moldova and north-eastern Romania
Source: PLoS Negl Trop Dis. 2023 Jul 6;17(7):e0011446. doi: 10.1371/journal.pntd.0011446 (PMC10325106; doi:10.1371/journal.pntd.0011446)
Supplement: S6 Table — (DOCX) [file pntd.0011446.s006.docx]

**S6 Table. Metadata associated with the sequences used for the Bayesian phylogenetic analysis of the partial N gene of rabies virus.**

| No. | Country | Region | Isolate | Species | Year of isolation | Phylogenetic group | GenBank  accession number | Reference |
| --- | --- | --- | --- | --- | --- | --- | --- | --- |
| 1 | Romania | Bacau | RO-RV-40-08-BC | Red fox  *(Vulpes vulpes)* | 2008 | NEE | GU086652 | [1] |
| 2 | Romania | Vrancea | DR1019 | Red fox  *(Vulpes vulpes)* | 2014 | NEE | OL449093 | This study |
| 3 | Romania | Vaslui | DR1187 | Red fox  *(Vulpes vulpes)* | 2014 | NEE | OL515144 | This study |
| 4 | Romania | Vrancea | DR1022 | Cow  *(Bos taurus)* | 2013 | NEE | OL449094 | This study |
| 5 | Romania | Bacau | DR1025 | Red fox  *(Vulpes vulpes)* | 2012 | NEE | OL449095 | This study |
| 6 | Romania | Iasi | DR1335 | Red fox  *(Vulpes vulpes)* | 2016 | NEE | OL515145 | This study |
| 7 | Moldova | Cimislia | DR1350 | Cow  *(Bos taurus)* | 2016 | NEE | OL515149 | This study |
| 8 | Moldova | Nisporeni | DR1349 | Cow  *(Bos taurus)* | 2016 | NEE | MW177594 | This study |
| 9 | Moldova | Calarasi | DR1201 | Cow  *(Bos taurus)* | 2016 | NEE | OL515146 | This study |
| 10 | Moldova | Chisinau | DR1198 | Goat  *(Capra aegagrus hircus)* | 2016 | NEE | OL515150 | This study |
| 11 | Moldova | Ialoveni | DR1357 | Ferret  *(Mustela putorius furo)* | 2016 | NEE | OL515148 | This study |
| 12 | Moldova | Straseni | DR1356 | Cow  *(Bos taurus)* | 2016 | NEE | OL515147 | This study |
| 13 | Romania | Botosani | DR1334 | Cow  *(Bos taurus)* | 2016 | NEE | OL515143 | This study |
| 14 | Romania | Iasi | DR1030 | Cow  *(Bos taurus)* | 2013 | NEE | OL449090 | This study |
| 15 | Moldova | Edineti | DR1347 | Cow  *(Bos taurus)* | 2016 | NEE | OM203139 | This study |
| 16 | Romania | Botosani | DR1336 | Cow  *(Bos taurus)* | 2016 | NEE | OM203140 | This study |
| 17 | Moldova | Criuleni | DR1200 | Dog  *(Canis lupus familiaris)* | 2016 | NEE | OM021440 | This study |
| 18 | Romania | Vaslui | DR1017 | Cat  *(Felis catus)* | 2014 | NEE | MW177595 | This study |
| 19 | Romania | Galati | DR1031 | Cow  *(Bos taurus)* | 2015 | NEE | OL440112 | This study |
| 20 | Moldova | Criuleni | DR1352 | Cow  *(Bos taurus)* | 2016 | NEE | OM203142 | This study |
| 21 | Moldova | Criuleni | DR1345 | Cow  *(Bos taurus)* | 2016 | NEE | OM203141 | This study |
| 22 | Moldova | Dondiuseni | DR1351 | Cat  *(Felis catus)* | 2016 | NEE | MW177593 | This study |
| 23 | Romania | Suceava | DR1332 | Cow  *(Bos taurus)* | 2016 | NEE | OL515142 | This study |
| 24 | Romania | Suceava | DR1333 | Red fox  *(Vulpes vulpes)* | 2016 | NEE | OL515141 | This study |
| 25 | Romania | Neamt | DR1033 | Red fox  *(Vulpes vulpes)* | 2013 | NEE | OL515133 | This study |
| 26 | Romania | Neamt | DR1036 | Dog  *(Canis lupus familiaris)* | 2013 | NEE | OL515135 | This study |
| 27 | Romania | Neamt | DR1034 | Red fox  *(Vulpes vulpes)* | 2013 | NEE | OL515134 | This study |
| 28 | Romania | Neamt | DR1032 | Dog  *(Canis lupus familiaris)* | 2012 | NEE | OL515136 | This study |
| 29 | Romania | Bacau | DR1027 | Roe deer  *(Capreolus capreolus)* | 2012 | NEE | OL515139 | This study |
| 30 | Romania | Neamt | DR1035 | Cow  *(Bos taurus)* | 2012 | NEE | OL515140 | This study |
| 31 | Romania | Bacau | DR1024 | Red fox  *(Vulpes vulpes)* | 2012 | NEE | OL515137 | This study |
| 32 | Romania | Bacau | DR1026 | Dog  *(Canis lupus familiaris)* | 2012 | NEE | OL515138 | This study |
| 33 | Romania | Satu Mare | RO-RV-2535-06-SM | Red fox  *(Vulpes vulpes)* | 2006 | NEE | GU086628 | [1] |
| 34 | Romania | Suceava | RO-RV-61-07-SV | Red fox  *(Vulpes vulpes)* | 2007 | NEE | GU086623 | [1] |
| 35 | Romania | Vrancea | DR1021 | Wolf  *(Canis lupus)* | 2014 | NEE | OL449092 | This study |
| 36 | Romania | Vrancea | DR1020 | Red fox  *(Vulpes vulpes)* | 2013 | NEE | OL449091 | This study |
| 37 | Moldova | Comrat | DR1348 | Cat  *(Felis catus)* | 2017 | NEE | OM203138 | This study |
| 38 | Moldova | Cahul | DR1343 | Cat  *(Felis catus)* | 2016 | NEE | OM203137 | This study |
| 39 | Moldova | Causeni | DR1353 | Cow  *(Bos taurus)* | 2016 | NEE | OM203136 | This study |
| 40 | Romania | Vrancea | DR1331 | Red fox  *(Vulpes vulpes)* | 2016 | NEE | OM021441 | This study |
| 41 | Romania | Ialomita | RO-RV-1281-06-IL | Dog  *(Canis lupus familiaris)* | 2006 | NEE | GU086632 | [1] |
| 42 | Romania | Bucuresti | RO-RV-63-07-B | Red fox  *(Vulpes vulpes)* | 2007 | NEE | GU086625 | [1] |
| 43 | Romania | Ialomita | RO-RV-1279-06-IL | Red fox  *(Vulpes vulpes)* | 2006 | NEE | GU086630 | [1] |
| 44 | Romania | Gorj | RO-RV-43-08-GJ | Red fox  *(Vulpes vulpes)* | 2008 | D | GU086654 | [1] |
| 45 | Romania | Gorj | RO-RV-46-08-GJ | Red fox  *(Vulpes vulpes)* | 2008 | D | GU086657 | [1] |
| 46 | Romania | Neamt | RO-RV-57-06-NT | Wolf  *(Canis lupus)* | 2006 | D | GU086616 | [1] |
| 47 | Romania | Mures | RO-RV-31-07-MS | Red fox  *(Vulpes vulpes)* | 2007 | D | GU086646 | [1] |
| 48 | Romania | Mures | RO-RV-28-07-MS | Red fox  *(Vulpes vulpes)* | 2007 | D | GU086643 | [1] |
| 49 | Romania | Gorj | RO-RV-45-08-GJ | Red fox  *(Vulpes vulpes)* | 2008 | D | GU086656 | [1] |
| 50 | Romania | Gorj | RO-RV-44-08-GJ | Red fox  *(Vulpes vulpes)* | 2008 | D | GU086655 | [1] |
| 51 | Romania | Arges | RO-RV-1302-06-AG | Cow  *(Bos taurus)* | 2006 | D | GU086631 | [1] |

* NEE: North-Eastern Europe; D: Centre of the European part of Russia

**REFERENCE**

1. Turcitu MA, Barboi G, Vuta V, Mihai I, Boncea D, Dumitrescu F, et al. Molecular epidemiology of rabies virus in Romania provides evidence for a high degree of heterogeneity and virus diversity. Virus Res. 2010;150(1–2).
