# Supplementary figures and images for: Whole genome sequencing and phylogenetic characterisation of rabies virus strains from Moldova and north-eastern Romania
Source: PLoS Negl Trop Dis. 2023 Jul 6;17(7):e0011446. doi: 10.1371/journal.pntd.0011446 (PMC10325106; doi:10.1371/journal.pntd.0011446)

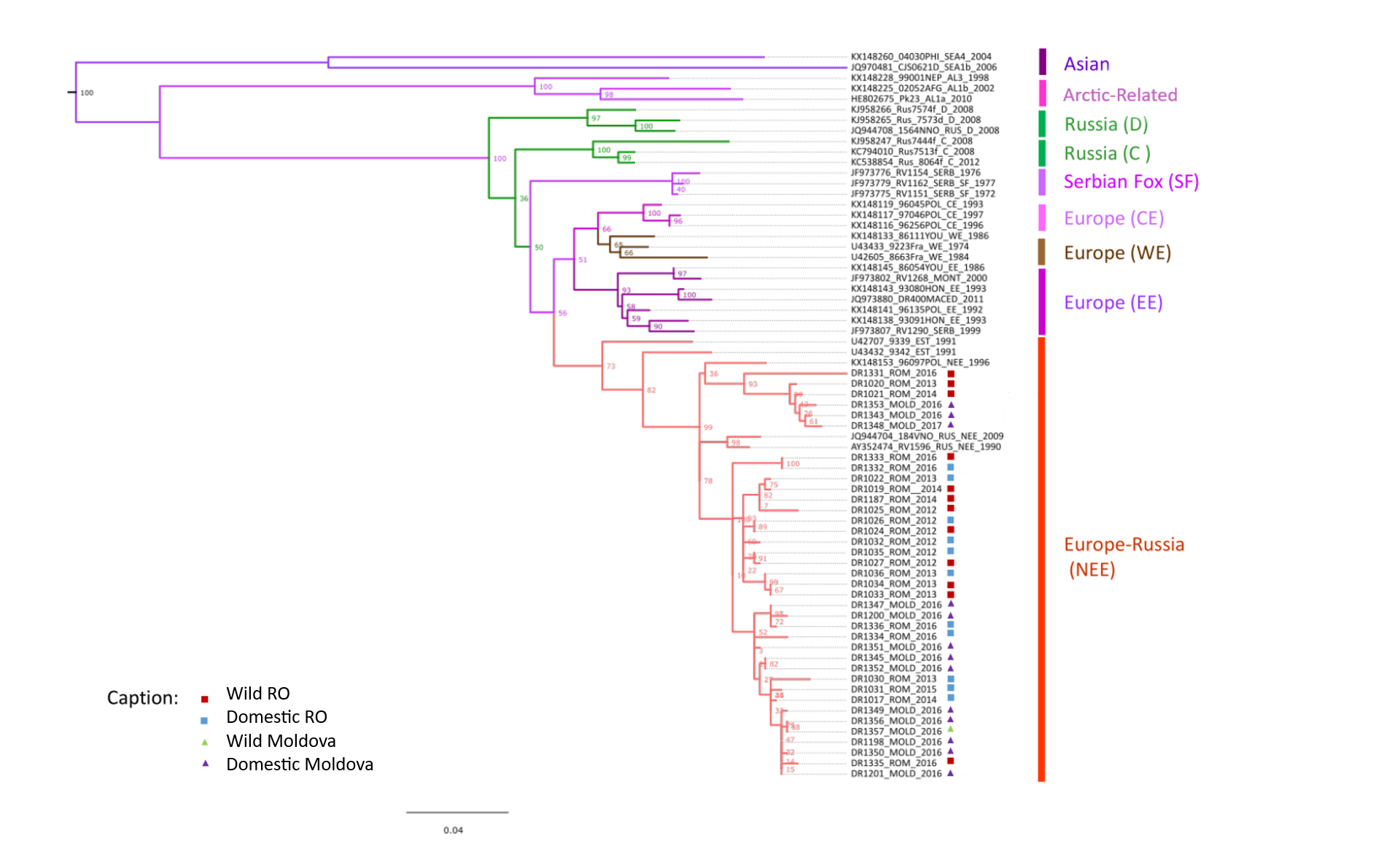

Supplement: S1 Fig — For Moldova, domestic animals (n = 13) are shown in purple triangles and the wild animal (n = 1) in a green triangle. For north-eastern Romania, domestic animals (n = 11) are shown in blue squares and wild animals (n = 12) in red squares. (TIFF) [file pntd.0011446.s007.tiff]
